# Changes in the immune landscape of TNBC after neoadjuvant chemotherapy: correlation with relapse

**DOI:** 10.3389/fimmu.2023.1291643

**Published:** 2023-11-09

**Authors:** Mohammed Ridha Moamin, Richard Allen, Steven Leslie Woods, Janet Elizabeth Brown, Harry Nunns, Anna Juncker-Jensen, Claire Elizabeth Lewis

**Affiliations:** ^1^ Division of Clinical Medicine, School of Medicine & Population Health, Faculty of Health, Sheffield, United Kingdom; ^2^ Neogenomics Labs., Aliso Viejo, CA, United States

**Keywords:** triple negative breast cancer (TNBC), multiplex optical bioassays, encoding, bioimaging and biodection, diagnostics, neoadjuvant chemotherapy (NAC), macrophages, TIM-3

## Abstract

**Introduction:**

Patients with high-risk, triple negative breast cancer (TNBC) often receive neoadjuvant chemotherapy (NAC) alone or with immunotherapy. Various single-cell and spatially resolved techniques have demonstrated heterogeneity in the phenotype and distribution of macrophages and T cells in this form of breast cancer. Furthermore, recent studies in mice have implicated immune cells in perivascular (PV) areas of tumors in the regulation of metastasis and anti-tumor immunity. However, little is known of how the latter change during NAC in human TNBC or their impact on subsequent relapse, or the likely efficacy of immunotherapy given with or after NAC.

**Methods:**

We have used multiplex immunofluorescence and AI-based image analysis to compare the immune landscape in untreated and NAC-treated human TNBCs. We quantified changes in the phenotype, distribution and intercellular contacts of subsets of tumor-associated macrophages (TAMs), CD4+ and CD8+ T cells, and regulatory T cells (Tregs) in PV and non-PV various areas of the stroma and tumor cell islands. These were compared in tumors from patients who had either developed metastases or were disease-free (DF) after a three-year follow up period.

**Results:**

In tumors from patients who remained DF after NAC, there was a marked increase in stromal CD163+ TAMs, especially those expressing the negative checkpoint regulator, T-cell immunoglobulin and mucin domain 3 (TIM*-*3). Whereas CD4+ T cells preferentially located to PV areas in the stroma of both untreated and NAC-treated tumors, specific subsets of TAMs and Tregs only did so only after NAC. Distinct subsets of CD4+ and CD8+ T cells formed PV clusters with CD163+ TAMs and Tregs. These were retained after NAC.

**Discussion:**

Quantification of stromal TIM-3+CD163+ TAMs in tumor residues after NAC may represent a new way of identifying patients at high risk of relapse. PV clustering of immune cells is highly likely to regulate the activation and function of T cells, and thus the efficacy of T cell-based immunotherapies administered with or after NAC.

## 1 Introduction

Triple negative breast cancer (TNBC) is a type of breast cancer that lacks oestrogen and progesterone receptors, as well as human epidermal growth factor receptor 2 (HER2). TNBC is associated with earlier age of onset, aggressive clinical course, rapid relapse after treatment, and worse prognosis compared to hormone receptor positive and HER2-positive breast carcinomas (1). TNBC also has more limited treatment options, with the mainstay being chemotherapy administered in the neoadjuvant and/or adjuvant/metastatic settings. Neoadjuvant chemotherapy (NAC) is being increasingly used for patients with high-risk TNBC but the majority fail to show a complete pathological response and relapse within three years (2). In an attempt to increase response rates and patient survival, attention has now turned to the use of immune checkpoint inhibitors (ICIs) in combination with NAC. Indeed, various clinical trials have showed that the addition of an ICI to NAC reduces relapse in some patients (3, 4).

Robust biomarkers are needed to predict relapse after NAC and inform patient selection for treatment with ICIs. Despite considerable effort to characterise the molecular and cellular features of TNBCs, very few have led to changes in treatment or improved outcomes for patients (5). So, there is now considerable focus on how the immune landscape of TNBCs is altered by NAC, and how this might help to predict subsequent relapse. Changes in this could also help to explain why some patients respond well to NAC administered with ICIs, while others do not.

Various studies have used single-cell transcriptomics and proteomics to demonstrate the presence of multiple subsets of immune effector cells like T cells and tumor-associated macrophages (TAMs) in human TNBCs (6–9). More recently, spatially resolved methods like digital spatial profiling, imaging mass cytometry, multiplexed ion beam imaging, and multiplex immunofluorescence have started to map important features of the immune landscape in this type of breast cancer. These studies have shown the importance of local signals and intercellular interactions in the regulation of immune cell function in specific compartments within tumors (10, 11). For example, they highlighted marked differences in immune cell functions in the tumor stroma versus the tumor cell islands (‘TCIs’, sometimes referred to as tumor ‘nests’) (12, 13).

There is also increasing evidence in mouse tumor models that some immune cells like TAMs, T cells and regulatory T cells (Tregs) may accumulate around tumor blood vessels, in what is often termed the ‘perivascular niche’ (PVN, usually defined as within 50µm from the abluminal surface of vessels) (14). This alters their pattern of gene expression and function(s) due to their exposure to endothelial-derived factors like angiopoietin-2 and interleukin-6 (15–17). For example, perivascular (PV) TAMs inhibit anti-tumor immunity and stimulate tumor angiogenesis and metastasis (14, 18). Furthermore, they have been shown to limit the efficacy of chemotherapy in mouse tumors, in part by blocking T cell recruitment across the tumor vasculature, and then promoting tumor angiogenesis and regrowth after the cessation of treatment (17, 19). This TAM subset also promote relapse by helping cancer cells to escape into the circulation during chemotherapy and form distant metastases (20, 21). However, recent evidence has emerged for functional diversity amongst PV TAMs as some appear to be able to augment rather than inhibit T-cell-mediated immunity in tumors (22, 23).

CD4+ T cells and Tregs have also been shown to accumulate around blood vessels in human glioma, and that this is an independent predictor of relapse-free survival (24). Additionally, a subset of CD4+ T cells aggregate around blood vessels in human breast carcinomas and correlate with poor prognosis (25). When it comes to the impact of PV CD8+ T cells, this appears to be dependent on cancer type as high numbers of these cells correlate with improved disease-free (DF) survival in resected hepatocellular carcinomas (26), but not in metastatic melanoma (27).

How the density, distribution and function of such immune cells as TAMs, Tregs, and T cells are altered in human TNBCs by NAC have yet to be fully defined. So, in this study we have used multiplex immunofluorescence and AI-based image analysis to analyse these features for the following four immune cell types in the stroma and TCIs of human TNBCs in untreated versus NAC-treated tumors: (i) CD163+ TAMs, (ii) CD3+CD4+FOXP3+ T regs, (iii) CD3+CD4+FOXP3- (‘CD4+’) T cells, and (iv) CD3+CD8+ (‘CD8+’) T cells. We included an assessment of PV and non-PV sites in these areas to allow PV immune changes to be defined for the first time.

Our data indicate that all four immune cell types studied showed the phenomenon of ‘immune exclusion’ as they were found mainly in the tumor stroma in both untreated and NAC-treated TNBCs. Of note, PV TAMs, T cells and Tregs often made direct contact with one another to form distinct, three-cell clusters. We discuss the implications of these observations for relapse after NAC alone, and the likely success of combining ICIs with chemotherapy in the neoadjuvant setting. We also identified a specific subset of TAMs which, when increased during NAC, correlated with the absence of metastasis over a three year follow up period.

## 2 Material and methods

### 2.1 Human TNBCs

FFPE sections (3µm) from 36 primary human TNBCs (age, size and grade-matched, anonymised) were from the Breast Tissue Bank of the UK charity, Breast Cancer Now. These were collected at the Barts Health NHS Trust Hospitals and Nottingham University NHS Trust Hospital in the UK between 2011 and 2017. Nineteen of these were from patients who did not receive NAC before definitive surgery (the ‘untreated’ group) and the other seventeen were from those given NAC followed by surgery. Approximately half of each of these two patient groups developed distant metastases within three years of surgery. These follow-up data were used to correlate multiplex immunostaining results with the presence or absence of metastasis during this period (ie. the ‘+Mets’ or ‘disease-free’, ‘DF’, groups respectively) (**Supplementary Table 1**). Power calculations were performed using data from our previous human multiplex immunofluorescence studies to ensure that sufficient tumors were included in each of the above four groups.

### 2.2 Multiplex immunofluorescence staining

Prior to multiplexing, whole FFPE tumor sections were stained with H&E and examined by a pathologist to ensure the presence of malignant tumor. Sections were then multiplexed with a custom panel, including the 10 markers listed below, as described previously (28). This ‘MultiOmyx’ procedure involved baking sections at 65°C for 1h, deparaffinizing and treating them with a two-step antigen retrieval process. They were then blocked against nonspecific binding with 10% donkey serum and 3% BSA in PBS for 1h at RT and stained with DAPI for 15 min. Directly conjugated primary antibodies (list below) were diluted in PBS supplemented with 3% (wt/vol) BSA (to working concentrations optimized previously) and applied for 1h at RT on a Leica Bond III Stainer.

Antibodies used were: mouse anti-LAG-3 (17B4, LifeSpan Biosciences), mouse anti-PanCK (PCK26, Sigma-Aldrich/AE1, BioScience), mouse anti-CD31/PECAM-1 (89C2, Cell Signaling), mouse anti-CD3 (F7.2.38, Dako), rabbit anti-CD4 (EPR6855, Abcam; Cat# ab196372, RRID : AB_2889191), mouse anti-CD8 (C8/144B, Dako), mouse anti-FoxP3 (206D, BioLegend), rabbit anti-PD-1 (EPR4877, Abcam), rabbit anti-PD-L1 (SP142, Abcam), mouse anti-TIM-3 (polyclonal, R&D Systems) and mouse anti-CD163 (EDHu-1, Bio-Rad).

TAMs were identified using an antibody to CD163 rather than the commonly used alternative, CD68, as the latter has been shown to label other cells such as fibroblasts in human breast tumors (29). We also examined TAM expression of two negative checkpoint regulators (NCRs), PD-L1 (programmed cell death ligand 1) and TIM-3 (T cell immunoglobulin and mucin domain-containing protein 3) as these have been linked to improved relapse free survival and/or survival in untreated TNBC (30, 31). Furthermore, the effect of NAC on TAM expression of these two cell surface receptors has yet to be defined.

The density and distribution of three other immune cell types were also quantified: Tregs identified using CD3, CD4 and FOXP3; CD4+ T cells using CD3 and CD4 and FOXP3 negativity (herein called ‘CD4+ T cells’); and cytotoxic T cells using CD3 and CD8. Finally, the activation status of CD4+ and CD8+ T cells was defined using a combination of the above markers with the T cell activation marker, PD-1 (programmed cell death protein 1) and T cell exhaustion marker, LAG-3 (lymphocyte-activation gene 3) (32). So, non-activated (naïve) T cells are PD-1-; activated T cells, PD-1+LAG-3-; and exhausted T cells, PD-1+LAG-3+.

Several rounds of paired antibody staining were performed in sequence on each tumor section using the antibodies listed above. After each round of staining with two antibodies, high resolution images were collected from 20 regions of interest (ROIs) across viable tumor areas using a 20x objective on an IN Cell analyzer 2200 microscope (GE Healthcare Life Sciences). ROIs included both PanCK-rich areas (TCIs) and PanCK-negative areas (stroma). The mean value of a given parameter (eg. TIM-3) was then calculated for each tumor section using these 20 ROIs, and the overall mean +/- SEM calculated for each tumor group using these values.

In between exposure to paired antibodies, slides were washed in PBS/0.3% TritonX-100 and dye inactivation performed by immersion in an alkaline solution containing H2O2 for 15 min with gentle agitation at room temperature. Slides were washed again in PBS, imaged to check the efficacy of the dye in-activation, and stained with the next pair of antibodies.

### 2.3 Quantitative image analysis

**Figure 1:**
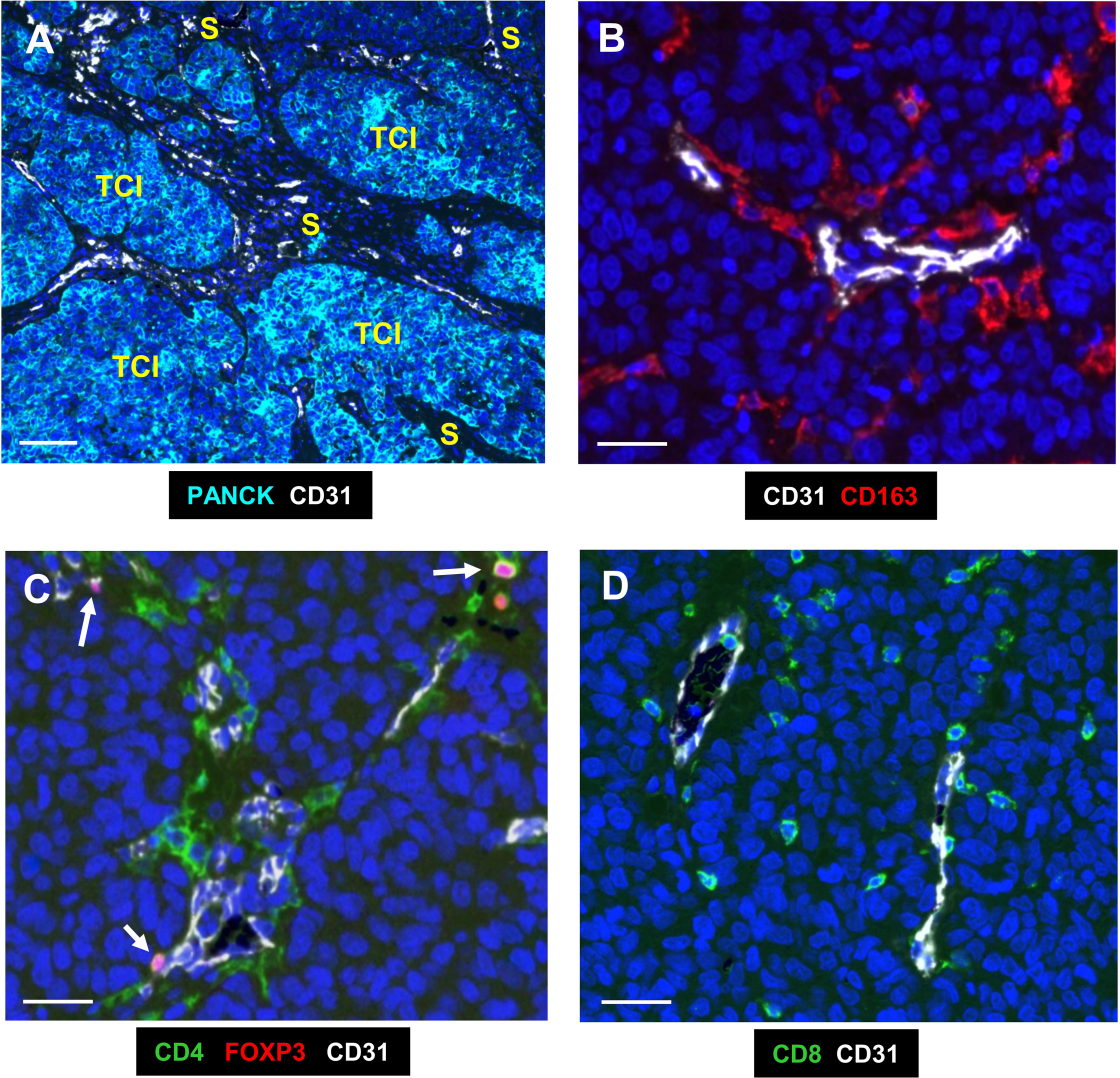
Representative fluorescence images of the two main compartments and immune cells analysed in human TNBC. **(A)** The 2 main compartments, tumor cell islands (TCIs) containing PANCK+ cancer cells, and the stroma which has most of the CD31+ blood vessels; white). **(B)** CD163+ TAMs (red) in the PV niche (PVN), the area within 50µm from the abluminal surface of a blood vessel. **(C)** CD4+ T cells (green) were mainly around CD31+ blood vessels, with some also expressing nuclear FOXP3. These are Tregs (pink; arrows). **(D)** CD8+ T cells (green) were more widely distributed across the stroma. Blue = DAPI staining of nuclei. Bar in **(A)** = 200 µm; bars in **(B–D)** = 50 µm.

An AI-based, advanced analytics platform, proprietary to NeoGenomics Labs, called ‘NEO Image Analysis’, was used to quantify and analyse subsets of TAMs, T cells and Tregs in various tumor areas. This included algorithms that could differentiate between them in stromal and TCIs, and within PV (within 50µm from a CD31+ blood vessel) and non-PV areas (>50µm) of TCIs and the tumor stroma (**Figure 1A**). Cells were segmented and tracked through each staining round, and deep learning models used to quantify positivity for each stain, as well as to classify regions as TCIs or stroma. The density of each cell type was calculated in each region of interest ‘ROI’ (for both PV and non-PV areas of the stroma versus TCIs) by dividing the total cell count in a given area by the area itself (in mm^2^).

Cell positivity for each marker was assessed based on detectable signal above background. Marker-specific AI algorithms were trained to incorporate both stain intensity and stain morphology in order to differentiate between true stain and nonspecific background or image artifacts. All analytical results (for every single image for each stain) were checked by a trained scientist to ensure concordance between the algorithms and scientist.

### 2.4 Statistical analysis

All data shown are means+/-SEMs and were analysed using GraphPad Prism (RRID : SCR_002798). Data analysis was done blind (ie with the four groups only identified after each analysis). Statistical analysis between groups was performed using the Mann-Whitney U-test with P values of ≤ 0.05 considered to be significant. All p values were corrected for multiple testing using the Bonferroni test. Pearson’s correlation analysis was used to assess the correlation between two groups of data.

The receiver operating characteristic (ROC) curve analysis graphically represents the ability of an observation to predict an outcome. It is a plot of the true positive rate (the rate at which the test accurately predicts the outcome), versus the false positive rate (the rate at which the test predicts the outcome incorrectly), at various threshold values of the observation. The performance of a test is often expressed as the Area Under Curve (AUC), which has a value of 1.0 for a test which is both 100% accurate and totally sensitive. An AUC of >0.7 is considered a “good” test result, whereas 0.5 or less indicates a test which gives random results.

## 3 Results

### 3.1 Distribution of CD163+ TAMs and their correlation with metastasis after NAC.

**Figure 2:**
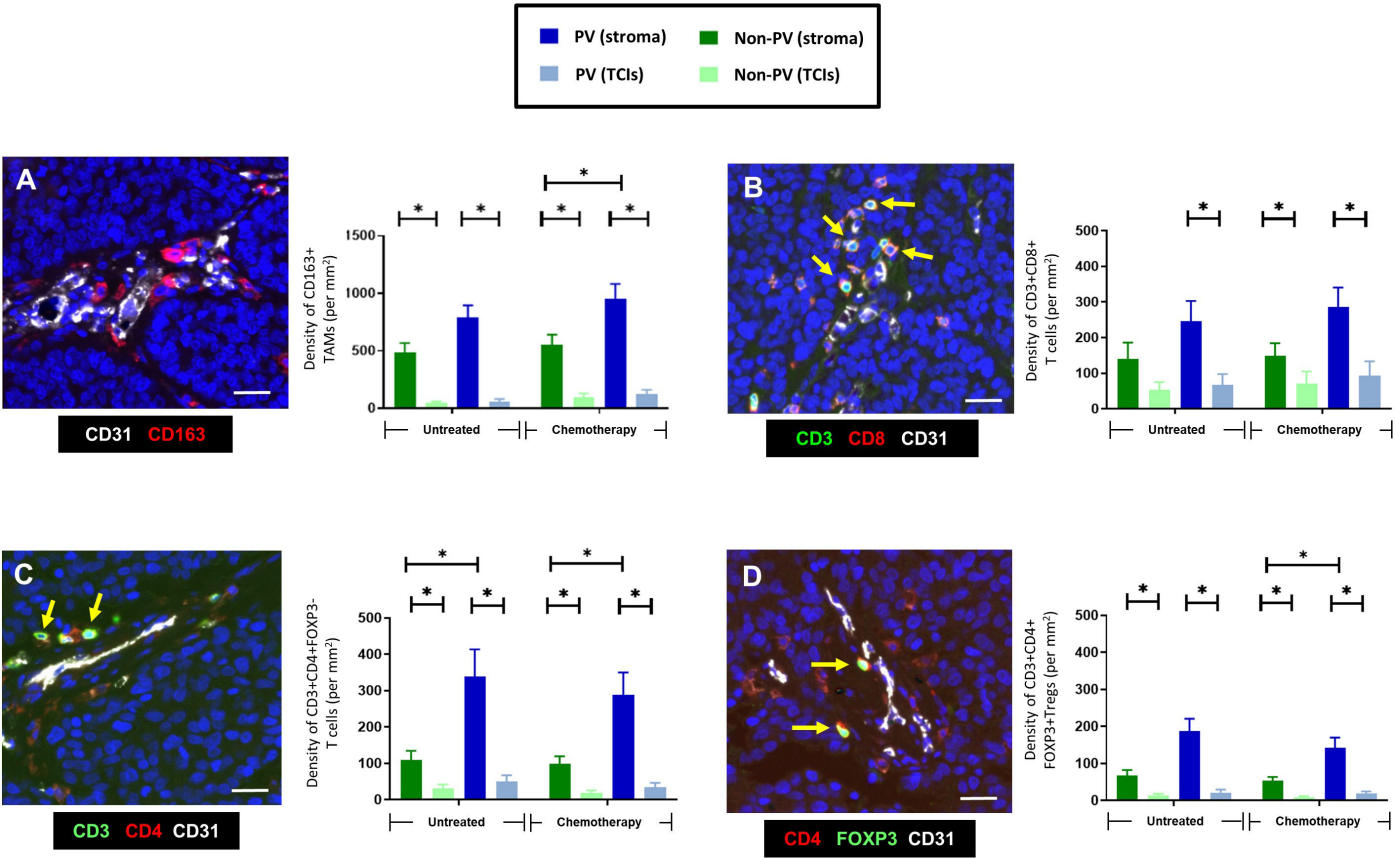
Frequency and distribution of CD163+TAMs, CD4+ and CD8+T cells, and Tregs. **(A)** Representative appearance of CD163+TAMs (red) relative to blood vessels in the tumor stroma between TCIs (left panel). The highest density of CD163+ TAMs was in the stroma, mainly in PV areas (right panel). **(B)** Representative appearance of CD3+CD8+ T cells relative to a small blood vessel in the tumor stroma (left panel; yellow arrows). This T cell subset was also located mainly to the stroma. **(C)** Representative appearance of CD3+CD4+ T cells relative to blood vessels in the tumor stroma (left panel; yellow arrows). CD3+CD4+FOXP3- T cells were mainly in PV areas of the stroma. **(D)** Representative appearance of CD3+CD4+FOXP3+ Tregs relative to blood vessels in the tumor stroma (left panel; yellow arrows). These cells were also mainly in PV areas of the stroma. [NB. In all groups in bar graphs, the stromal density was significantly higher than the corresponding TCI area but asterices were not shown for clarity]. Blue = DAPI staining of nuclei. *P<0.05. Bars = 50 µm.

**Figure 1A** illustrates the two main compartments analysed in this study; the TCIs in which cancer cells were labelled using a panCK antibody, and the stroma in between them. Both CD31+ blood vessels and the 4 groups of immune cells assessed (CD163+TAMs, CD4+ and CD8+ T cells and CD4+FOXP3+Tregs) were present mainly in the stroma (**Figures 1**, **2**). As mentioned previously, the PV area is defined as being within 50µm of the abluminal surface a given CD31+ blood vessel (8). It excludes the area of CD31 staining for blood vessels as well as their lumens.

CD163+ TAMs (**Figures 1B**, **2A**) and Tregs (**Figures 1D**, **2D**) were preferentially located in PV areas of the stroma after NAC. By contrast, CD4+ T cells were mainly PV in untreated as well as NAC-treated tumors (**Figures 1C**, **2C**), and CD8+T cells were evenly distributed across the stroma (**Figures 1D**, **2B**). The density of PV and non-PV CD163+ TAMs showed a trend towards a reduction in the stroma of the NAC +Mets group compared to the NAC DF group although this achieved significance only in the non-PV group (**Supplementary Figure 1A**). Links with metastasis were not seen with the other three immune cell types studied, although stromal T regs were significantly (p= 0.01) lower in the PV ‘+Mets’ group after NAC than the PV +Mets group in untreated tumors (**Supplementary Figures 1B–D**).

### 3.2 Most CD163+ TAMs lack expression of PD-L1

**Figure 3:**
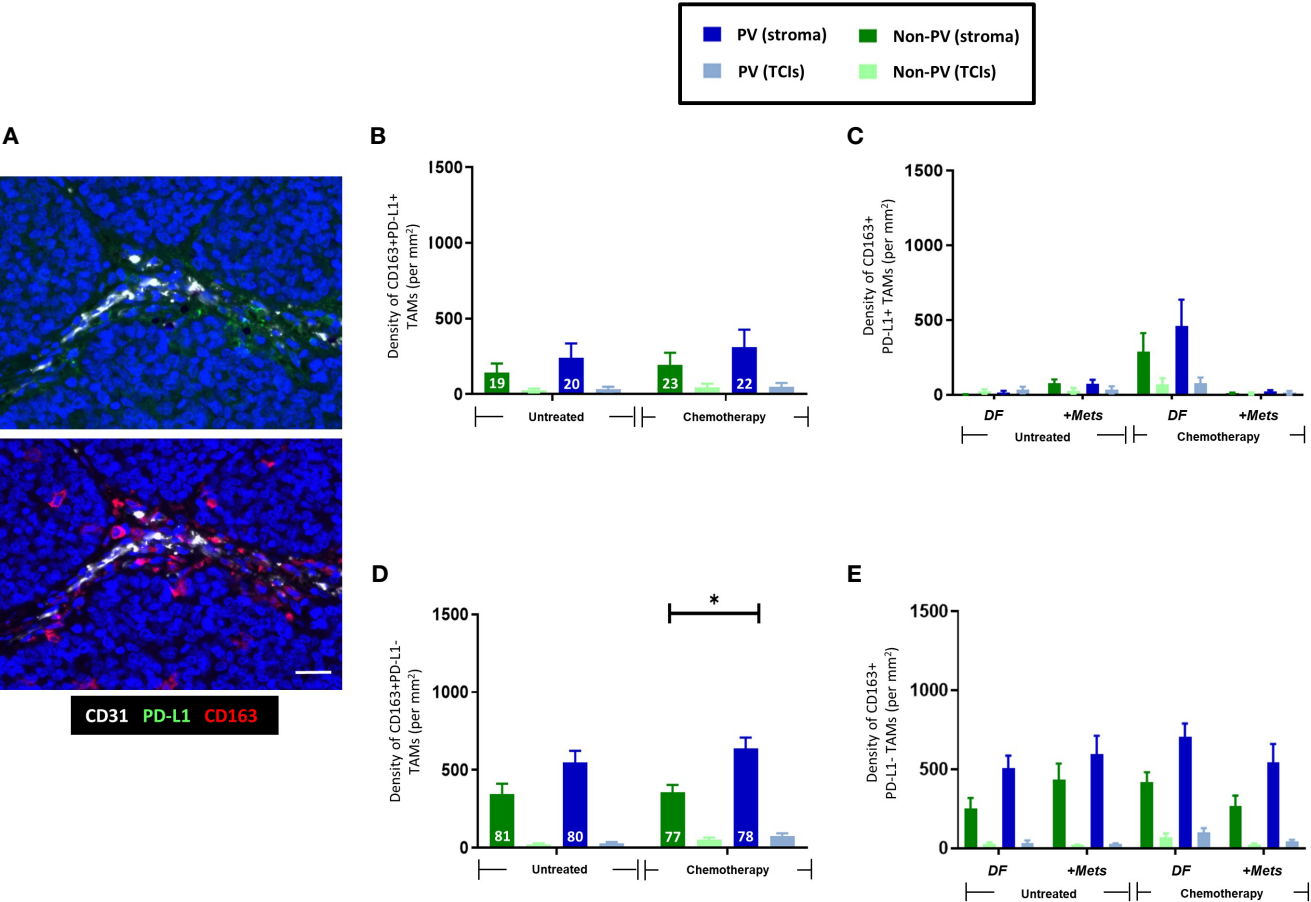
Expression of PD-L1 by CD163+TAMs. **(A)** Representative appearance of sparse stromal PD-L1 staining (top panel; green) and greater density of CD163+ TAMs in the same area (bottom panel; red) in the tumor stroma. Bar = 50 µm. The majority of stromal CD163+ TAMs do not express PD-L1. Blue = DAPI staining of nuclei. **(B)** PD-L1+CD163+ TAMs are evenly distributed across the stroma and generally exist at lower density than PD-L1-CD163+ TAMs **(D)**. **(C)** The density of PV PD-L1+CD163+ TAMs exhibited a non-significant trend towards being lower in PV areas of the stroma in the ‘+Mets’ group than the DF one after NAC, a feature not seen with PD-L1-CD163+ TAMs **(E)**. **(D)** Unlike PD-L1+ CD163+TAMs, PD-L1- CD163+ TAMs preferentially locate to PV areas after NAC. The white figures at the base of each bar are the % of CD163+ TAMs in each group that were either PD-L1+ (B) or PD-L1- (D). [NB. In all groups in bar graphs, the stromal density was significantly higher than the corresponding TCI area but asterices for this were not shown for clarity]. *P<0.05 (ns = not statistically significant).

In this study, the majority of TNBCs were found to contain at least some cancer cells and CD163+ TAMs expressing detectable PD-L1 although there was considerable heterogeneity in this between tumors (**Supplementary Figures 2A, B, D**). When we examined PD-L1 expression specifically by CD163+ TAMs, the majority (77-80%) of stromal CD163+TAMs were found to lack detectable PD-L1 expression and to be preferentially located in PV areas in the stroma after NAC (**Figures 3A, B, D**). By contrast, CD163+ TAMs that expressed PD-L1 were evenly distributed across the stroma and unaffected by NAC. Neither PD-L1+ nor PD-L1- CD163+TAMs correlated with metastasis (**Figures 3C, E**).

### 3.3 TIM3+CD163+ TAMs increased after NAC in tumors from patients who did not develop metastases

**Figure 4:**
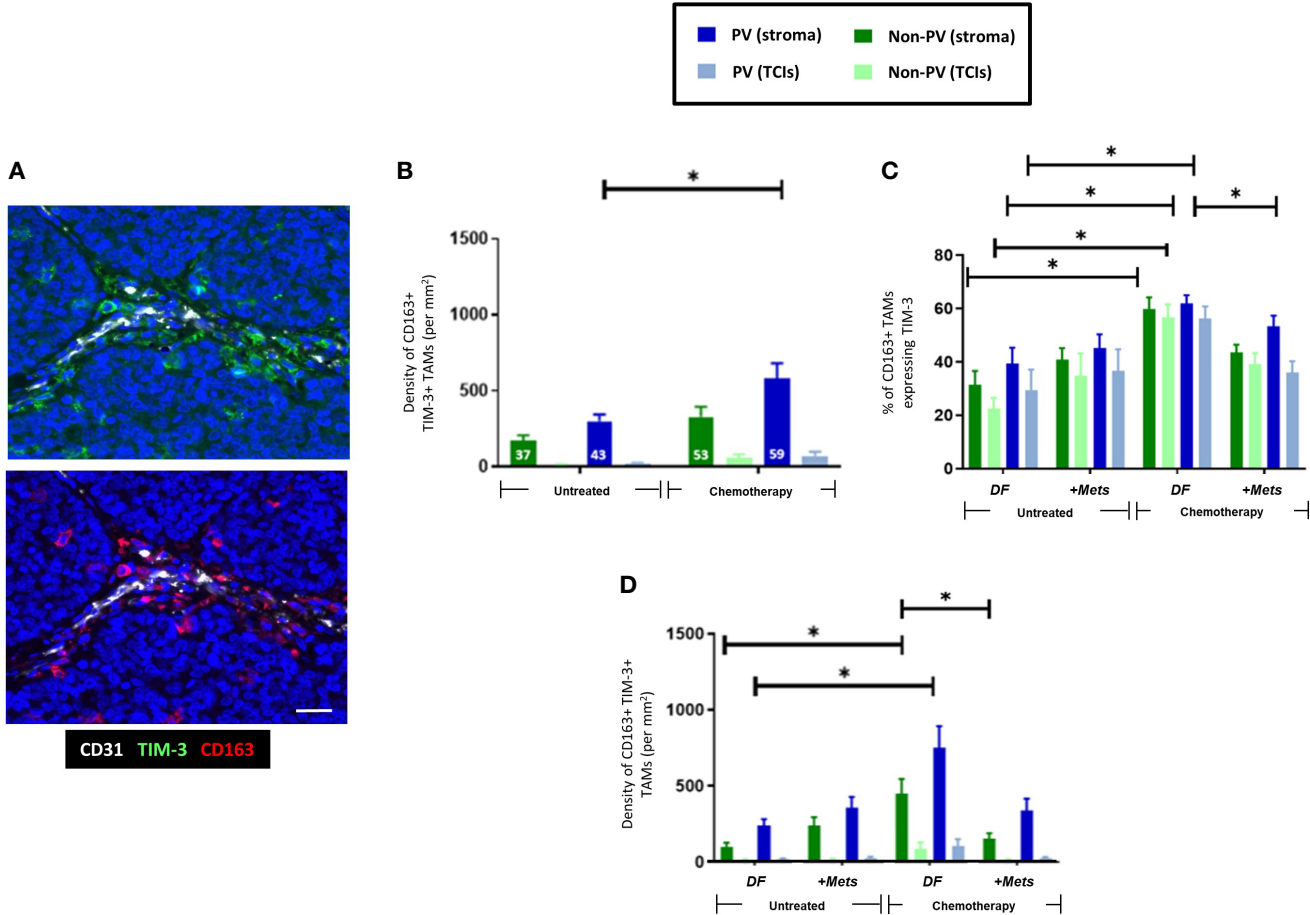
Upregulation of TIM-3 expression by CD163+ TAMs after NAC. **(A)** Representative appearance of TIM-3 (top panel) and CD163 (bottom panel) staining relative to blood vessels in the tumor stroma (yellow arrows highlight TIM-3+CD163+ TAMs). Blue = DAPI staining of nuclei. Bar = 50 µm. **(B)** The density of TIM-3+CD163+ TAMs was elevated in PV areas of the stroma after NAC. The white figures at the base of each bar are the % of CD163+ TAMs expressing TIM-3 in that group. **(C)** NAC increased the % of CD163+ TAMs expressing TIM-3 in the DF but not the ‘+Mets’ group. **(C)** TIM-3+CD163+ TAMs were distributed evenly across the stroma in all groups and significantly reduced in all areas after NAC in the ‘+Mets’ group compared to the DF group. **(D)**. The density of TIM-3+CD163+ TAMs was elevated in the stroma of the DF but not the ‘+Mets’ group after NAC. [NB. In all groups in panels **(A, D)** the stromal density was significantly higher than the corresponding TCI area but asterices were not shown for clarity]. *P<0.05.

As with CD163+ TAMs (**Figure 2A**), NAC induced an increase in PV TIM-3+CD163+TAMs (**Figures 4A, B**). There was also a significant (p=0.008) increase in the proportion of CD163+TAMs in all tumor areas expressing TIM-3 after NAC in the DF group compared to the untreated, DF group (**Figure 4C**), Taken together these two findings suggest that NAC leads to TIM-3 upregulation by existing CD163+ TAMs in the DF group and/or the recruitment of TIM-3-expressing TAMs into the stroma in the DF group. **Figure 4D** also shows that, after NAC, the DF group contained a significantly (p=0.0015) higher density of non-PV TIM-3+CD163+ TAMs than in non-PV areas of the +Mets group. None of the above features were seen with TIM-3-CD163+ TAMs (**Supplementary Figure 3**).

To interrogate the ability of stromal CD163+ TAMs or TIM3+CD163+ TAMs to predict relapse in NAC-treated TNBC patients, we then conducted ROC curve analyses of the density of these two TAM subsets (DF v ‘+Mets’). We also included an analysis of TIM-3-CD163+ TAMs for comparison. These analyses showed that both CD163+ and TIM-3+CD163+ TAMs were strongly predictive of metastasis (area under the curve, ‘AUC’, values: CD163+ TAMs, 0.8143 (p=0.0318); TIM-3+CD163+ TAMs, 0.800 (p=0.0404)). (**Supplementary Figures 4A–C**). We also demonstrated that this predictive power resided in both PV or PV subsets CD163+ and TIM-3+CD163+ TAMs (ie. in cells across the entire stroma) (**Supplementary Figures 4D–F**). By contrast, the AUC value for TIM-3-CD163+ TAMs showed a weaker predictive power with metastasis, 0.6857 (p=0.2046). The latter accords well with the absence of any significant differences in TIM-3-CD163+ TAMs between the NAC DF and +Mets groups (**Supplementary Figure 3B**).

Although TIM-3 is known to be expressed by various cell types in human tumors our study demonstrated that CD163+ TAMs were the main cell type expressing this protein in TNBCs (**Supplementary Figure 5A**). Our finding that TNBC patients with high levels of stromal TIM-3-expressing CD163+ TAMs after NAC were less likely to develop metastasis within three years was supported, albeit inferentially, by data from a previously published dataset. These showed that when 153 TNBC patients were divided into those with high or low TIM-3 expression after NAC, the latter group had significantly worse relapse free survival (**Supplementary Figure 5B**) (33).

**Figure 5:**
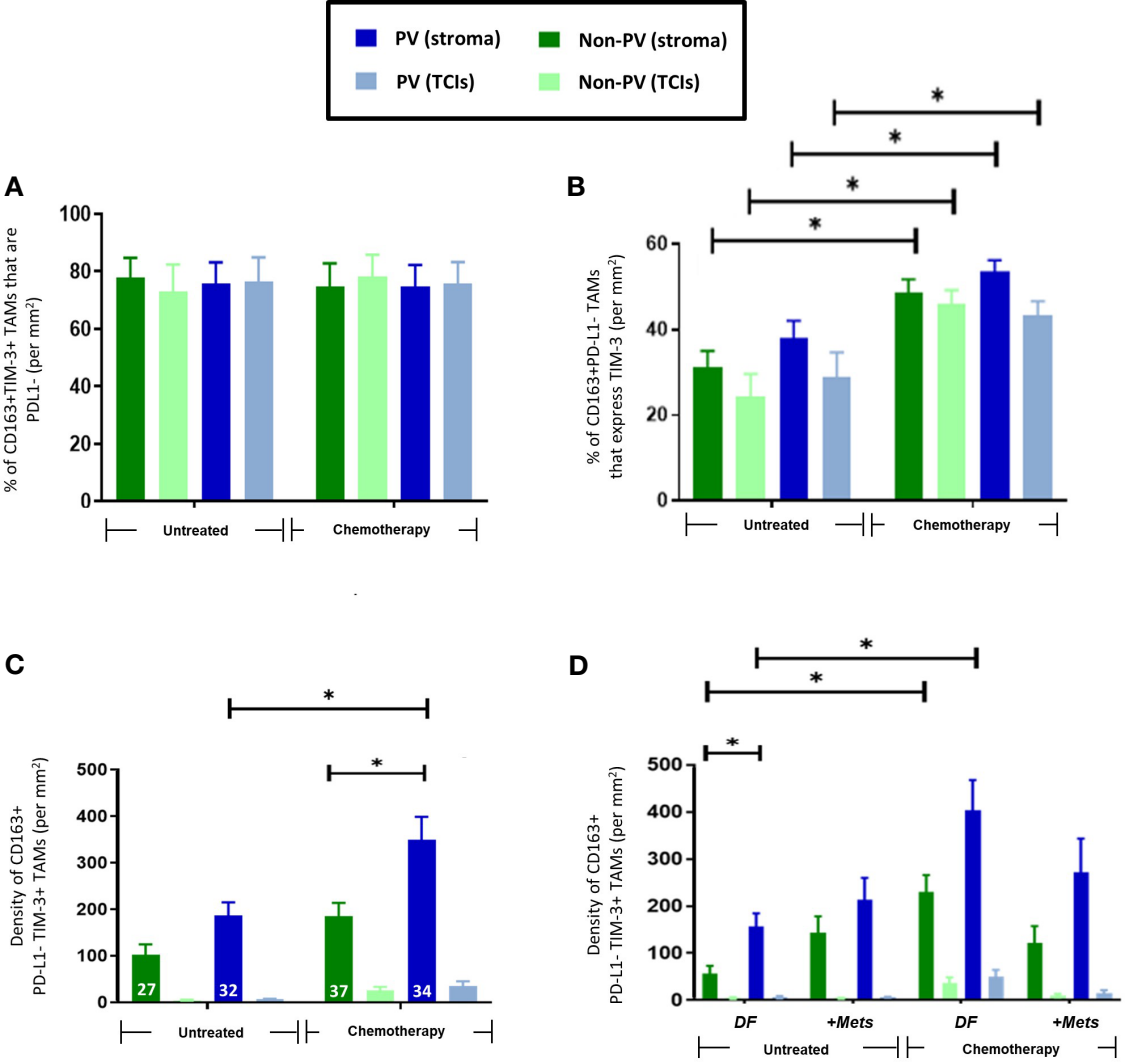
Frequency and distribution of PD-L1-TIM-3+CD163+TAMs. **(A)** 75-85% of CD163+TIM-3+ TAMs in all tumor areas failed to express detectable PD-L1. **(B)** NAC significantly increased the % of PD-L1-CD163+TAMs expressing TIM-3 (throughout tumors). **(C)** PD-L1-TIM-3+TAMs were mainly PV and exhibited increased numbers in all areas of NAC-treated compared to untreated tumors. The white figures at the base of each bar are the % of CD163+ TAMs that were both PD-L1- and TIM-3+ in that group. **(D)** The increased density of this TAM subset after NAC occurred mainly in PV areas of both treatment groups. [NB. in all groups in panels **(B, C)** stromal PV and non-PV groups were significantly higher than corresponding groups in TCIs, asterices not shown for clarity]. P<0.05. [NB. None of the TAM features shown in panels **B–D** were exhibited by the 15-25% of CD163+TIM-3+ TAMs that expressed PD-L1 (data not shown)].

Interestingly, over 75% of TIM-3+CD163+TAMs were PD-L1- (**Figure 5A**) and NAC significantly (p=001) increased the proportion of PD-L1-CD163+TAMs expressing TIM-3 (**Figure 5B** **).** The density of PD-L1-TIM-3+CD163+ TAMs was significantly (p= 0.002, non-PV; p=0.005, PV) increased in stromal areas of tumors after NAC, being highest in PV areas (**Figures 5C, D**). This effect was restricted to this specific TAM subset as it did not occur in other CD163+TAM subsets: PD-L1+TIM-3+, PD-L1+TIM-3- or PD-L1-TIM-3- (**Supplementary Figure 6**). Of note, when TIM-3+CD163+ TAMs were divided up into PD-L1- or PD-L1+ (**Figure 5D**, **Supplementary Figure 6D**), the significant correlation of TIM3+CD163+ TAMs with metastasis was lost.

### 3.4 Effect of NAC on stromal PV Tregs

CD3+CD4+FOXP3+ Tregs located preferentially to PV areas of the stroma in both untreated (p=0.045) and NAC-treated (p=0.036) ‘+Met’ groups. However, their density in these PV areas was significantly (p=0.01) lower in the NAC +Mets’ group than in the untreated ‘+Mets’ group (**Supplementary Figure 1D**).

### 3.5 Activation status of CD4+ and CD8+ T cells in the PVN

**Figure 6:**
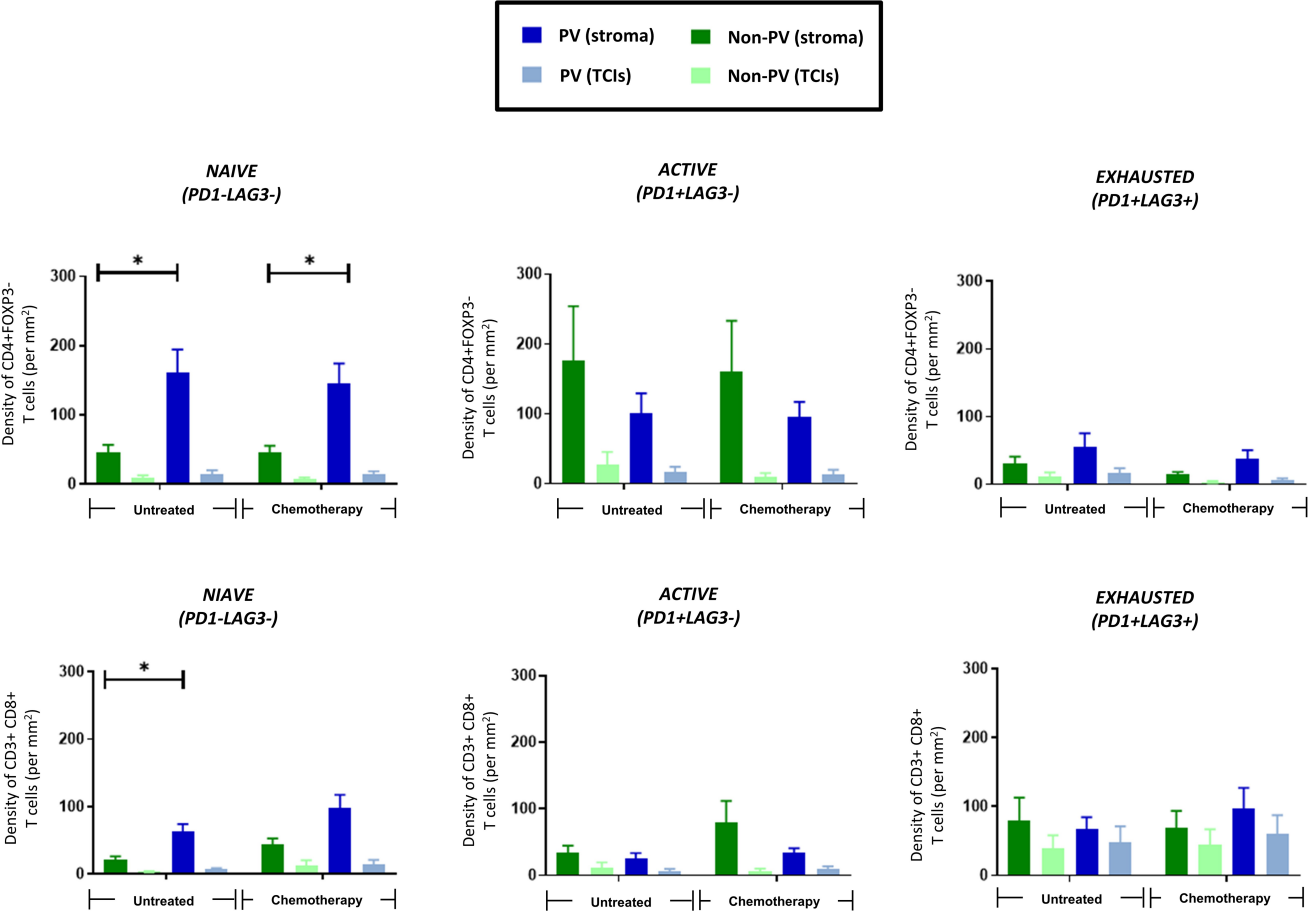
PV accumulation of naïve CD4+ and CD8+ T cells. The distribution of 3 subsets of CD3+CD4+FOXP3- T cells (top row) and CD3+CD8+ T cells (bottom row) are shown in PV v non-PV areas of the stroma and TCIs of untreated and NAC-treated tumors. Only naïve T cells preferentially accumulated in PV areas. *P<0.05.

The activation status of CD4+ and CD8+ T cells was assessed using antibodies to the activation marker, PD-1, and exhaustion marker, LAG-3 (ie. naïve T cells are PD1-LAG3-; active T cells are PD1+LAG3-; exhausted T cells are PD1+LAG3+). **Figure 6** shows that only naïve CD4+ (top row) and CD8+ T cells (bottom row) preferentially located to PV areas of the stroma in untreated tumors. This persisted in NAC tumors for CD4+ T cells. By contrast, both active and exhausted CD4+ and CD8+ T cells were evenly distributed across tumors (although active subsets of both CD4+ and CD8+ T cells showed a non-significant trend towards a more stromal distribution). Exhausted CD4+ T cells were only present at low density in all areas whereas exhausted CD8+ T cells showed greater variation between tumors with some containing a similar density in the stroma to naïve CD8+ T cells. Taken together, these data suggest that PV areas of tumors contain mainly naïve and active CD4+ T cells, and naïve and exhausted CD8+ T cells.

Interestingly, correlation analysis of the density of the above subsets of T cells (as well T regs) with the PD-L1+ and PD-L1- CD163+ TAM subsets showed that PD-L1+CD163+ TAMs showed a highly significant, positive correlation with all 3 activation subsets of CD4+ T cells, CD8+ T cells (and Tregs). This correlation was not seen with PD-L1-CD163+ TAMs (**Supplementary Table 2**, **Supplementary Figure 7**).

### 3.6 Perivascular clustering of distinct subsets of T cells with Tregs and CD163+ TAMs

**Figure 7:**
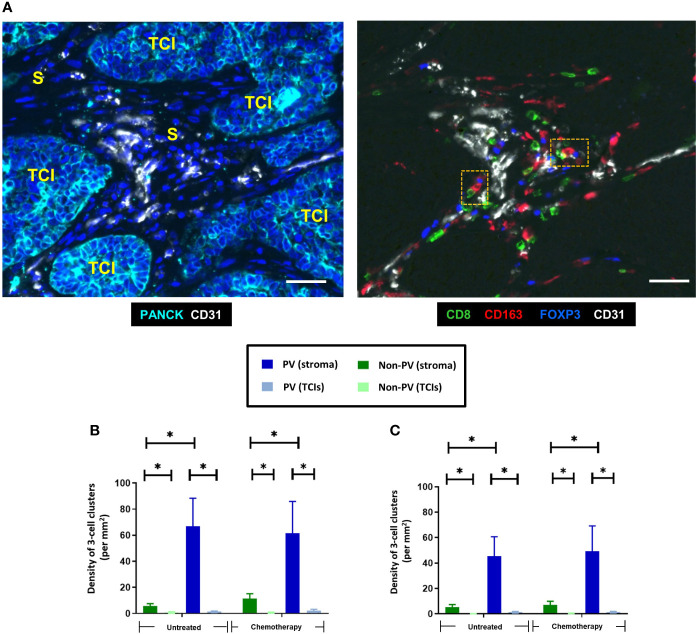
Distinct clusters of three immune cell types (CD163+ TAMs, CD8+ T cells and FOXP3+ Tregs) accumulate around blood vessels in both untreated and NAC-treated TNBCs. **(A)** Representative appearance of PV 3-cell clusters present around blood vessels in the stroma. Left panel: stromal areas (S) containing blood vessels are present between PANCK+ TCIs. Blue = DAPI staining of nuclei. Right panel: Close interaction between CD163+ TAMs, CD8+ T cells and FOXP3+ Tregs around blood vessels in the same tumor area shown in panel **(A)** The orange dashed boxes in the right panel show two, 3-cells clusters, each containing a CD163+ TAM, a CD8+ T cell and FOXP3+ Treg. Bars = 50 µm. Density of 3-cell clusters containing either CD4+ T cells **(B)** or CD8+ T cells **(C)**. They exist mainly in PV areas of the stroma. *P<0.05.

**Figure 8:**
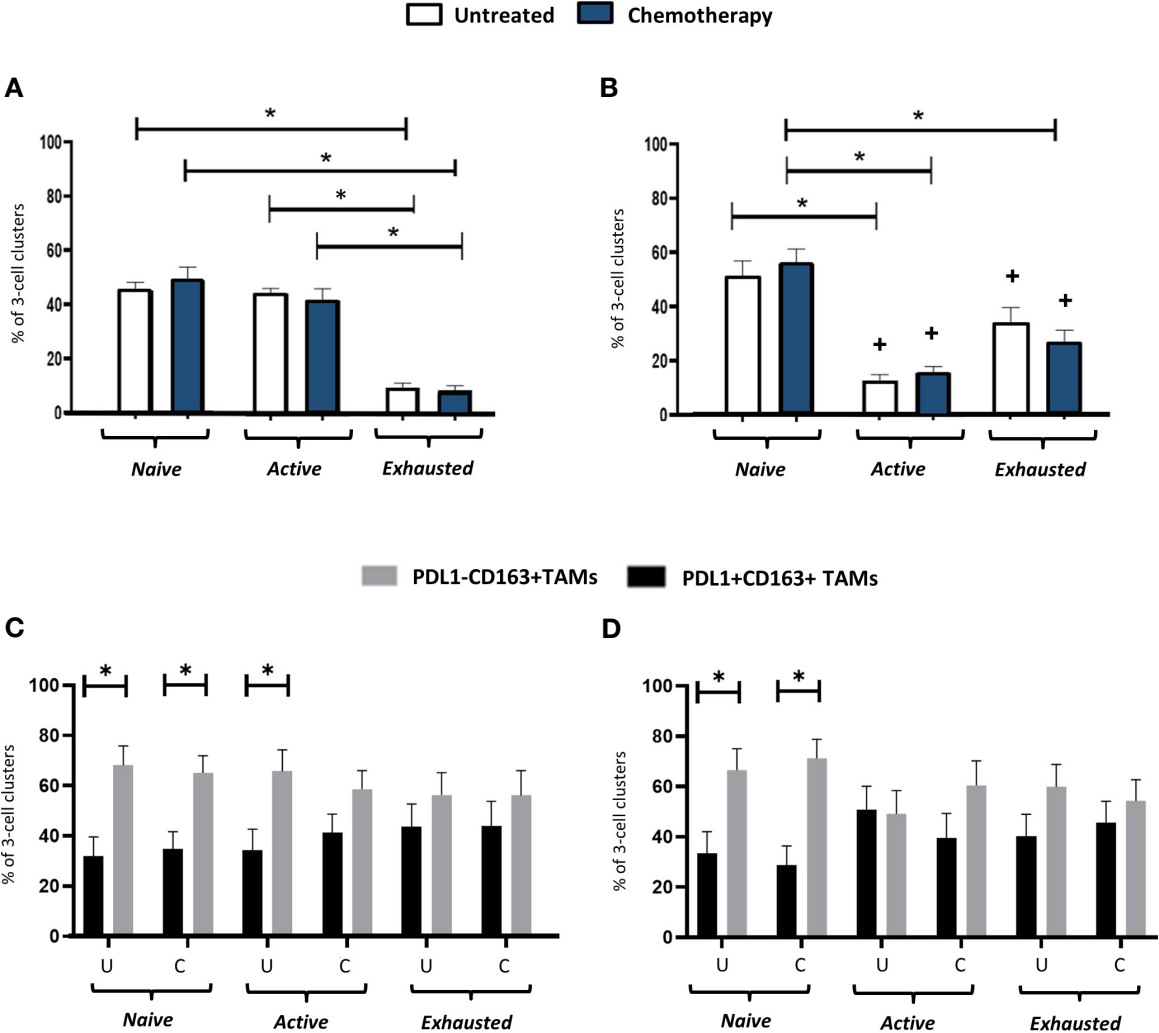
Activation status of T cells in PV, 3-cell clusters: correlation with PD-L1 status of CD163+ TAMs. The proportion of PV 3-cell clusters in the stroma containing either naïve, active or exhausted CD3+CD4+FOXP3- T cells **(A, C)** or a CD3+CD8+ T cells **(B, D)** (top row), or PD-L1- or PD-L1+ TAMs (bottom row). The ratio of TIM-3+:TIM-3- TAMs in PD-L1-TAMs in these clusters with 1:1 (data not shown). U, untreated; C, NAC-treated. *P<0.05. ^+^P<0.05 w.r.t the same group in **(A)**.

Distinct, 3-cell clusters were seen in the PV areas of the stroma of untreated and NAC-treated tumors. These consisted of a CD163+TAM, either a CD4+ (FOXP3-) or a CD8+ T cell and a CD4+FOXP3+ Treg (**Figure 7**). In keeping with the relatively high levels of naïve and active CD4+ T cells (along with naïve and exhausted CD8+ T cells) in the PV niche (**Figure 6**), the main types of PV 3-cell clusters were those containing naïve and active CD4+ and naïve and exhausted CD8+T cells (40-60%). The proportion of clusters containing active CD8+ T cells was significantly (p<0.0001) lower than those with active CD4+ T cells but the opposite was true when clusters contained exhausted T cells (**Figures 8A, B**). When PD-L1 expression by CD163+ TAMs in these PV clusters was interrogated, both PD-L1- and PD-L1+ ones were found to be present but significantly (p=0.002, untreated CD4; p=0.003, NAC CD4; p=0.003, untreated CD8; p=0.0004, NAC CD8) more PD-L1-CD163+ TAMs were present in clusters containing naïve T cells. This was also the case for active CD4+ T cells (p=0.008). (**Figures 8C, D**).

## 4 Discussion

Our finding that all four of the immune cell groups studied here reside mainly in the stroma of TNBC, accords well with other reports of such ‘immune exclusion’ from TCIs in both mouse and human tumors (34, 35). Indeed, several stromal cell types, such as TAMs, Tregs, MDSCs and cancer-associated fibroblasts (CAFs) have been shown to limit T cell infiltration into TCIs (35). Although high levels of T cells in the stroma correlate with improved survival in breast cancer (36), their accumulation away from TCIs also limits the efficacy of T cell-based immunotherapies, as cytotoxic T cells require close contact with cancer cells to kill them (37). So, the retention of T cells in the stroma after NAC is likely to limit the efficacy of immunotherapy administered with or after NAC.

Somewhat surprisingly, our data showed that high levels of stromal CD163+ TAMs in NAC-treated tumor residues correlated with the absence of metastasis within three years of primary surgery. This TAM subset also appeared to have utility as a predictive biomarker for metastasis in our ROC analyses, although this awaits validation in a larger, independent cohort of NAC-treated TNBCs. A number of studies have shown that high CD163+ TAMs in pre-NAC biopsies correlate with worse treatment responses (38, 39) but our data suggest that opposite is the case in post-NAC TNBCs. This is supported by the finding that high CD163+ TAMs in NAC-treated human pancreatic tumors correlate with improved disease-free progression compared to tumors with low levels (40).

When we then divided CD163+ TAMs by their TIM-3 expression we found that the link with metastasis resided with the TIM-3-expressing subset. This raises the possibility that this TAM subset is amplified in the stroma during NAC where it exerts an anti-metastatic effect. They could limit metastases by inhibiting the escape of cancer cells through tumor blood vessels and/or their ability to form metastases at distant sites. They could also phagocytose cancer cells and present their antigens to naïve T cells in PV tumor areas (or draining lymph nodes) to activate their tumoricidal functions. The latter could result in increased intra-tumoral or systemic immunity to metastasizing cancer cells.

At first glance, the link between this TAM subset and reduced metastasis appears to contradict the finding that high levels of TIM-3+ TAMs correlate with increased metastasis and/or reduced survival in other tumor types like untreated human non-small cell lung cancer and clear cell renal carcinomas (41, 42). However, the opposite appears to be the case for stromal TIM-3+ TILs (including TAMs) in untreated TNBC (31) so this appears to be context-dependent. Furthermore, to our knowledge the present study is the first to report specifically on the prognostic significance of stromal TIM-3+ TAMs after NAC.

Interestingly, cancer cell-derived TGFβ is known to stimulate the expression of both TIM-3 by TAMs (43, 44) and is upregulated by chemotherapeutic agents (45). However, it remains to be seen whether TGFβ contributes to the abundance and/or the possible anti-metastatic function(s) of stromal TIM-3+ TAMs, within the complex milieu of NAC-treated tumors.

Various immune cells were seen to preferentially accumulate in PV areas of TNBCs after NAC including CD163+ TAMs (both PD-L1- and TIM-3+), naïve CD4+ T cells and Tregs. This contrasted with CD4+ T cells which were also located mainly around blood vessels in untreated tumors, and CD8+ T cells that were widely distributed across the stroma under both conditions.

When it comes to PV TAMs, Arwert et al. (46) showed in a mouse model of mammary cancer that immature TAMs can be stimulated by cancer cells to upregulate CXCR4. This then causes them to traffic towards CXC12 expressed by fibroblasts around vessels. As mentioned earlier, various studies have reported that PV TAMs promote tumor resistance to chemotherapy, as well as tumor regrowth and metastatic spread after this treatment (14, 17–19, 21). As multiple subsets of macrophages are present in untreated tumors (6–8), the possibility of more than one TAM subset existing in PV areas untreated or NAC-treated TNBCs exists. Indeed, two recent studies have highlighted the presence of TAM subsets in the PVN with apparently opposing effects on tumor immunity. Sharma and colleagues (47) demonstrated the presence of a subset of PV, CD163hi TAMs that interact closely with T cells and Tregs in the PVN of human colorectal tumors and suppress T cell activation. This appears to be similar to our observation of these 3 cells forming PV clusters in TNBCs and suggests that they may inhibit anti-tumor immunity. Their presence in TNBCs after NAC could, therefore, limit the efficacy of ICIs given during or after NAC. However, it should be noted that Ramos et al. (22) recently described a subset of PV CD163+ that clustered at high density with CD8+ T cells, expressed genes that stimulate the cytotoxic function of T cells, and correlated with favourable clinical outcome in human breast cancer. The latter finding is supported by the demonstration that activated TAMs (as well as dendritic cells) colocalize with CD8+ T cells in mouse colorectal and pancreatic tumors, and that a high frequency of these PV TAM-T cell clusters correlated with improved tumor responses to immunotherapy (23). These latter findings raise the possibility that the PV clusters we describe could actually augment T cell activation and thus the efficacy of immunotherapy.

Using quantitative, multi-parameter imaging, such PV immune cell clusters in other mouse tumor modes have been shown to contain a specific subset of CD8+ T cells called ‘resource’ CD8+ T cells (ie. a subset of non-exhausted, PD-1-expressing cells with the capacity for enhanced proliferation) (23). It was proposed that the close interaction leads this subset to proliferate and rapidly develop into terminally differentiated, cytotoxic CD8+ T cells (23). This may accord with our finding that the main form of T (albeit CD4+) cells present in PV clusters in TNBCs were active (PD1+LAG3-) or naïve (ie. PD-1-) ones. The active ones may be analogous to the ‘resource T cells’ reported above in PV clusters in the mouse tumors (23). However, it should be noted that the above was not seen for CD8+ T cells in our cohort of TNBCs. In their case, the main form in clusters was naïve, with very few in an active state. The reason for the difference between CD4 and CD8+ cells is presently unclear.

It is possible that incorporation of naïve T cells into PV clusters is simply a way to retain them in an inactive state as soon as they enter tumors across the vasculature. It remains a possibility that these structures can regulate T cell function in a number of ways. Over 40% of PV clusters contained active CD4+ T cells which may have stemmed from naïve CD4+ T cells in clusters that were activated by antigen presented to them by CD163+ TAMs. Moreover, some or all of the Tregs present in clusters may have formed from naïve CD4+ T cells as the intra-tumoral generation of T regs from CD4+ T cells has recently been reported (48). It is noteworthy though that, in human breast cancer xenografts grown in humanized mice, blocking the recruitment of naïve CD4+ T cells into PV areas of tumors resulted in reduced numbers of intra-tumoral Tregs, and inhibited tumor progression (25). Maintaining newly formed Tregs (and/or those recruited from the peripheral blood) in PV clusters close to other immune effectors in TNBC would be likely to have a suppressive effect on the latter - unless PV Tregs are in an immature, non-functional state.

Our study shows that CD163+ TAMs in PV clusters containing either naïve or active CD4+ T cells, or naïve CD8+ T cells, lacked expression of PD-L1. Of note, the immunostimulatory PV TAM subset described recently in mouse colorectal tumors also lacked PD-L1 expression (23). However, further analysis of the phenotype of such ‘cluster TAMs’ is now needed to help understand the effect of such PV clusters on T-cell mediated immunity in TNBC.

Of course, it remains a possibility that T cells could also influence the phenotype of TAMs or Tregs in PV clusters. For example, naïve T are unable to release interferon (IFN)-γ (49), an important stimulus for PD-L1 expression by macrophages in tumors (50). This could help to explain to our finding that CD163+ TAMs in clusters with naïve T cells were predominantly lacking in PD-L1 expression.

Taken together, our data have identified a distinct subset of TAMs that correlates inversely with metastasis after NAC. As such, they could be a new biomarker for relapse. They also show that a number of important immune effectors form intimate contacts with one another in PV areas of TNBCs, a feature retained after NAC. These clusters are highly likely to impact on T cell function and thus the success of immunotherapy administered with NAC.

## Data availability statement

The original contributions presented in the study are included in the article/**Supplementary Material**. Further inquiries can be directed to the corresponding author.

## Ethics statement

The studies involving humans were approved by Breast Cancer Now’s ethics review committee. The studies were conducted in accordance with the local legislation and institutional requirements. The participants provided their written informed consent to participate in this study.

## Author contributions

MM: Conceptualization, Data curation, Formal Analysis, Methodology, Writing – review & editing. RA: Conceptualization, Formal Analysis, Investigation, Methodology, Writing – review & editing. SW: Formal Analysis, Writing – review & editing. JB: Formal Analysis, Writing – review & editing. HN: Data curation, Formal Analysis, Methodology, Software, Writing – review & editing. AJ: Conceptualization, Data curation, Formal Analysis, Funding acquisition, Methodology, Project administration, Software, Supervision, Writing – review & editing. CL: Conceptualization, Data curation, Formal Analysis, Funding acquisition, Investigation, Methodology, Project administration, Supervision, Validation, Visualization, Writing – original draft, Writing – review & editing.

## Supplementary material

Supplementary Figure 1 Reduced density of stromal CD163+ TAMs correlate inversely with metastasis. Distribution of CD163+ TAMs **(A)**, CD3+CD8+ T cells **(B)**, CD3+CD4+FOXP3- T cells **(C)** and CD3+CD4+FOXP3+ Tregs **(D)** in PV and non-PV areas of the stroma and TCIs in untreated and NAC-treated groups (subdivided into those that did or did not develop metastases within 3 years of primary surgery. ‘DF’ = DF; ie. no metastases; ‘+Mets’ = developed metastases). **P<0.05.*

Supplementary Figure 2 Representative appearance of PD-L1 expression by CD163+ TAMs and PANCK+ cancer cells in high/moderate PD-L1-expressing TNBCs. PD-L1+CD163+ TAMs were most frequent at the interface between TCIs and the stroma. Bar = 50 µm.

Supplementary Figure 3 Frequency and distribution of TIM-3-CD163+TAMs. **(A)** TIM-3-CD163+ TAMs were present throughout the stroma in untreated and NAC-treated tumors. The white figures at the base of each bar are the % of CD163+ TAMs in each group that were TIM-3-. **(B)** The density of this TAM subset did not correlate with metastasis. [NB. in all groups, stromal PV and non-PV groups were significantly higher than corresponding groups in TCIs, asterices not shown for clarity]. *P*<0.05.

Supplementary Figure 4 Receiver Operating Characteristic (ROC) curve analysis of the ability of three stromal TAM subsets to predict the development of metastasis in NAC-treated patients within 3 years of surgical removal of primary tumors (ROC analysis of data at the level of whole tumor sections). **(A)** all CD163+ TAMs; **(B)** all TIM3-3+CD163+ TAMs; **(C)** all TIM-3-CD163+ TAMs; **(D)** PV v non-PV CD163+ TAMs; **(E)** PV v non-PV TIM-3+CD163+ TAMs; **(F)** TIM-3-CD163+ TAMs.

Supplementary Figure 5 Expression of TIM-3 in human TNBC: correlation with relapse free survival after NAC. **(A)** CD163+ TAMs and alpha smooth muscle actin-positive cells (‘ASMA’; pericytes and fibroblasts) are the predominant cell types expressing TIM-3 - and the proportion of these cells expressing this cell surface protein increases after NAC. ASMA = alpha smooth muscle actin. **P<0.05.* **(B)** Significant correlation between TIM-3 expression levels in 153 TNBCs (taken from women administered NAC) and DFS (using online dataset: http://kmplot.com/analysis/index.php?p=service&cancer=breast#) (33).

Supplementary Figure 6 No effect of NAC on various CD163+ TAM subsets. Three CD163+ TAM subsets **(A, D)** PD-L1+TIM-3+; **(B, E)** PD-L1+TIM-3-; **(C, F)**. PD-L1-TIM-3) were evenly distributed throughout the stroma of tumors at significantly higher density than in corresponding areas of TCIs (asterices not shown). There was no effect of NAC on the density or distribution of these cells, nor did they correlate with metastasis. (ns = not statistically significant).

Supplementary Figure 7 Correlation graphs for significantly different comparisons seen in **Supplementary Table 2**. R = correlation coefficient.

Supplementary Table 1 Clinical parameters for the four patent groups analysed in this study. Parentheses indicate percentage data. NAC = neoadjuvant chemotherapy (3-8 cycles). DF = DF (no metastasis within 3 years of primary surgery), ‘+Mets’ = developed metastases within 3 years. FEC = 5 fluorouracil, epirubicin, cyclophosphamide. FEC-T - 5 = fluorouracil, epirubicin, cyclophosphamide, docetaxel. ‘Other’ NACs included FEC-T & Carboplatin, EC (epirubicin cyclophosphamide) and ECD (epirubicin cyclophosphamide & docetaxel).

Supplementary Table 2 Correlation of the overall density of PD-L1+ CD163+TAMs with CD4+ T cells, CD8+ T cells and Tregs.
