# Supplementary figures and images for: Changes in the immune landscape of TNBC after neoadjuvant chemotherapy: correlation with relapse
Source: Front Immunol. 2023 Nov 9;14:1291643. doi: 10.3389/fimmu.2023.1291643 (PMC10715438; doi:10.3389/fimmu.2023.1291643)

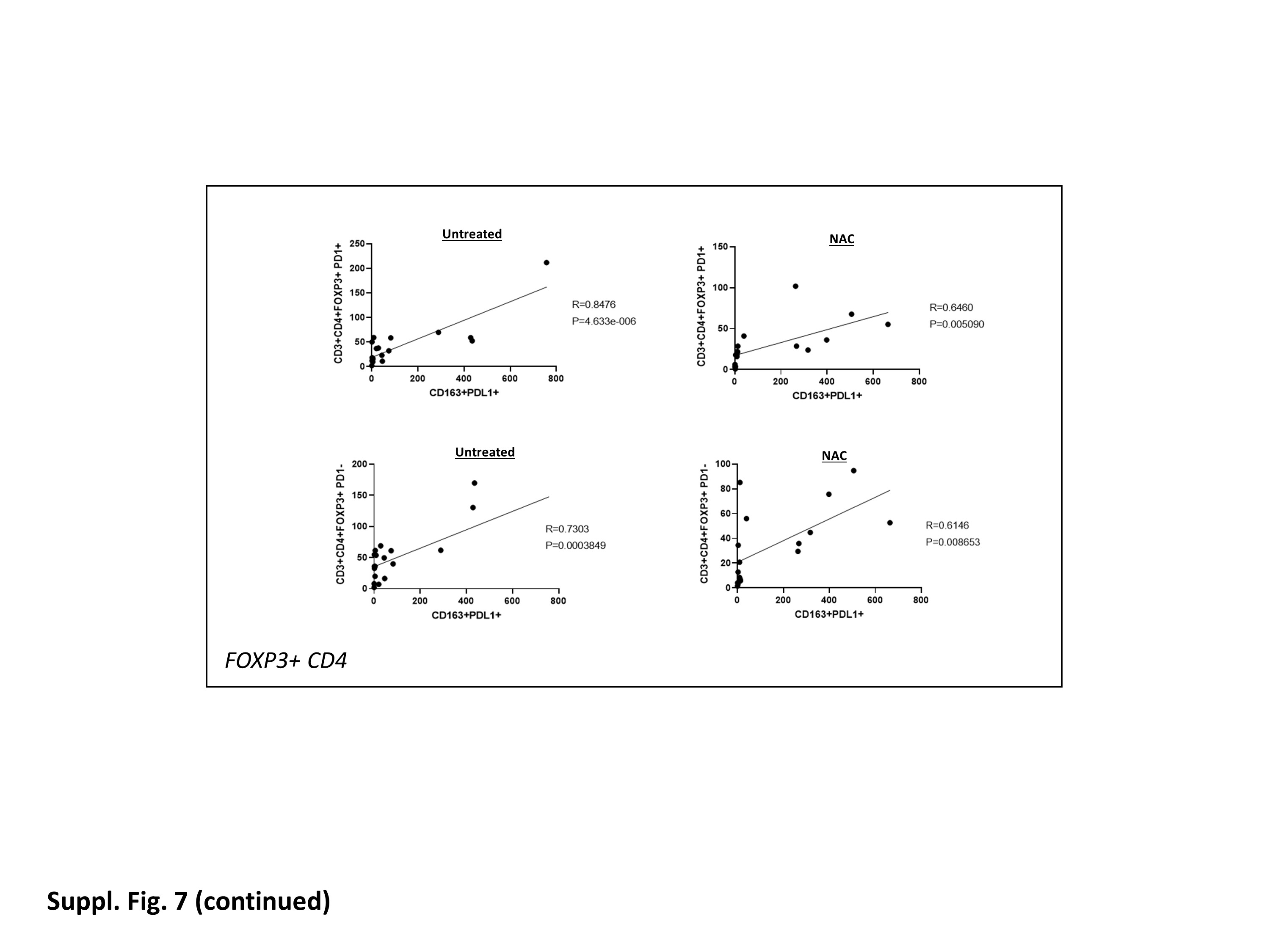

Supplement: Supplementary file 1 [file Image_8.jpg]

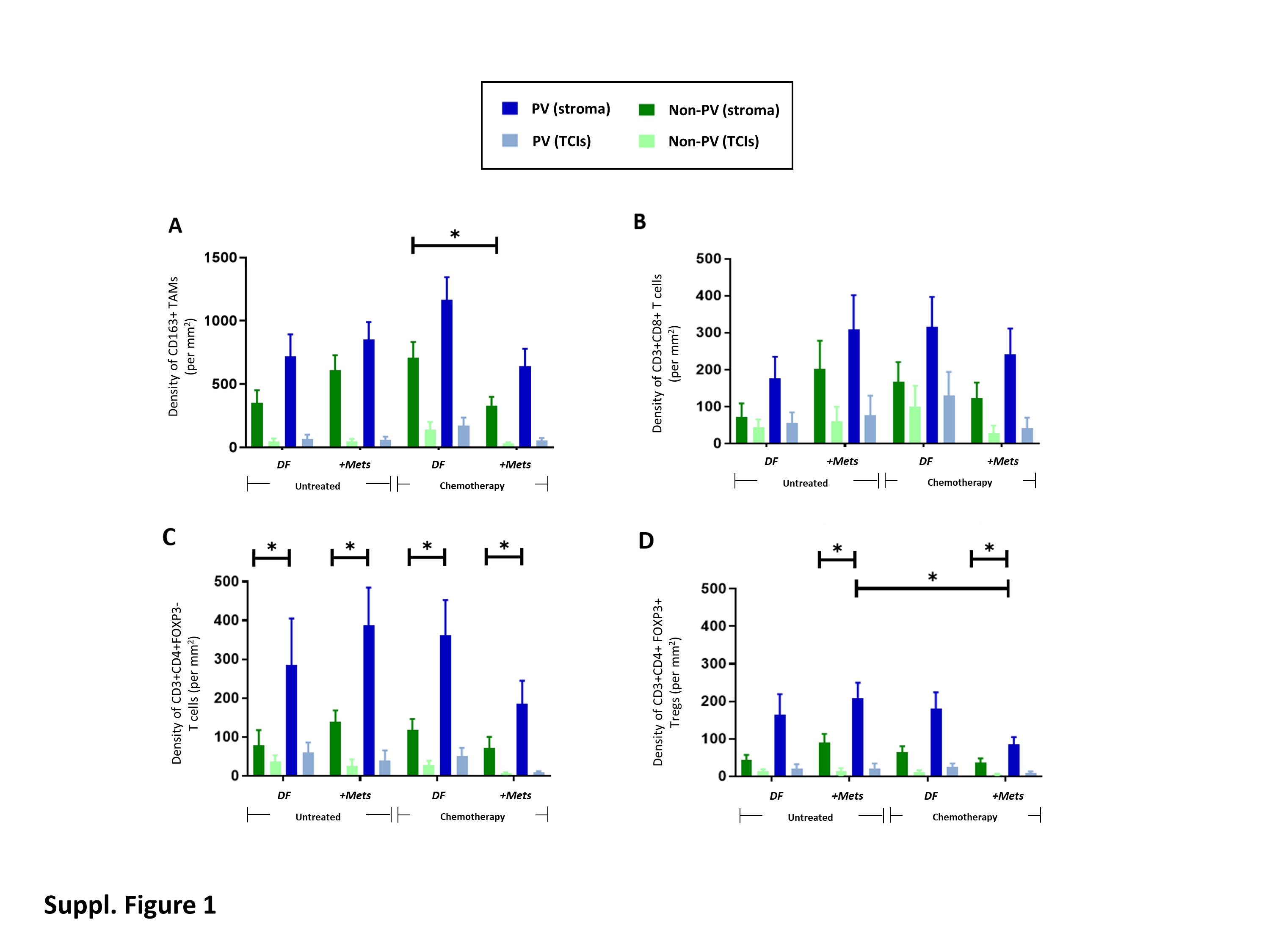

Supplement: Supplementary Figure 1 — Reduced density of stromal CD163+ TAMs correlate inversely with metastasis. Distribution of CD163+ TAMs (A), CD3+CD8+ T cells (B), CD3+CD4+FOXP3- T cells (C) and CD3+CD4+FOXP3+ Tregs (D) in PV and non-PV areas of the stroma and TCIs in untreated and NAC-treated groups (subdivided into those that did or did not develop metastases within 3 years of primary surgery. ‘DF’ = DF; ie. no metastases; ‘+Mets’ = developed metastases). *P<0.05. [file Image_1.jpg]

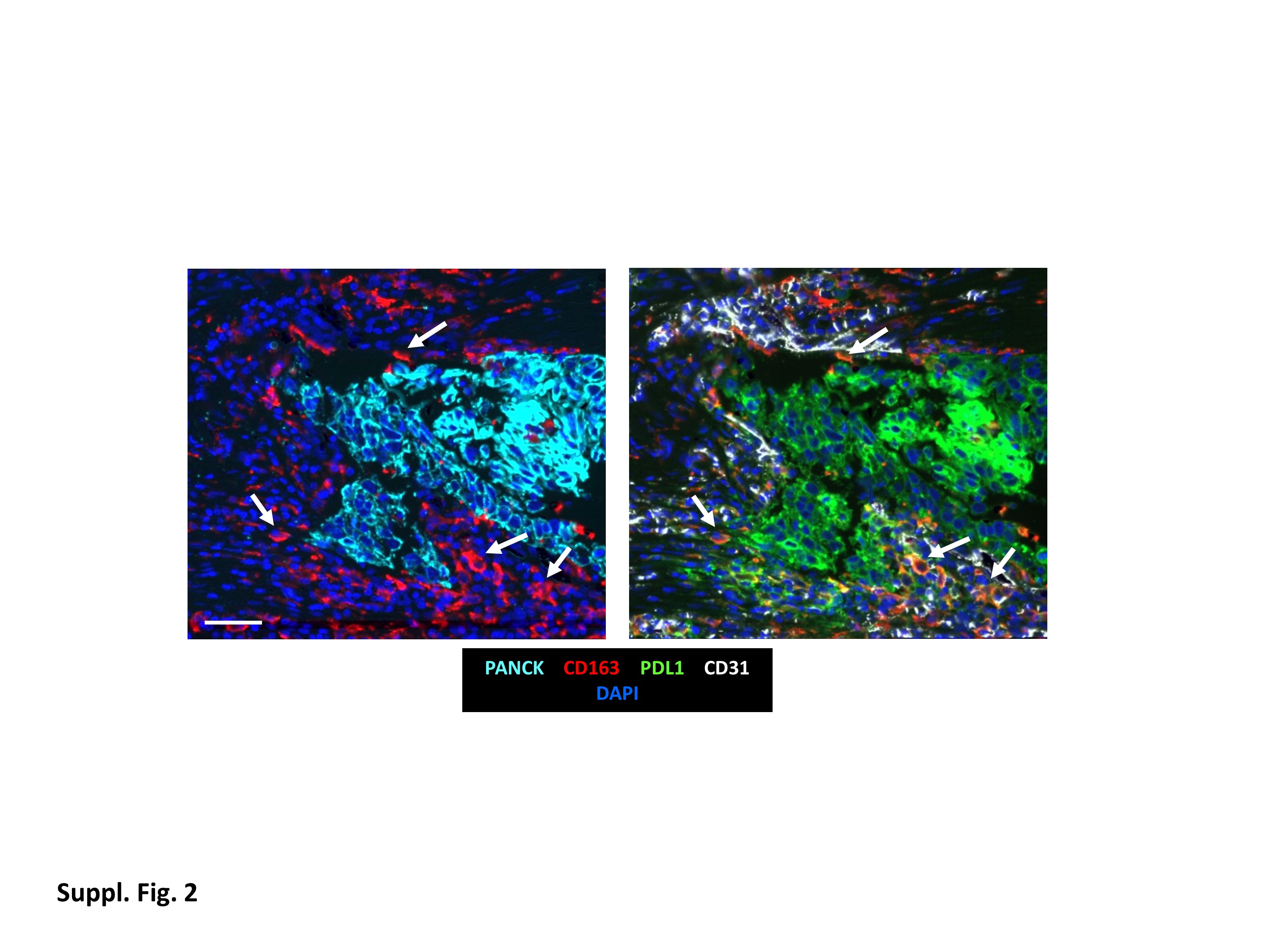

Supplement: Supplementary Figure 2 — Representative appearance of PD-L1 expression by CD163+ TAMs and PANCK+ cancer cells in high/moderate PD-L1-expressing TNBCs. PD-L1+CD163+ TAMs were most frequent at the interface between TCIs and the stroma. Bar = 50 µm. [file Image_2.jpg]

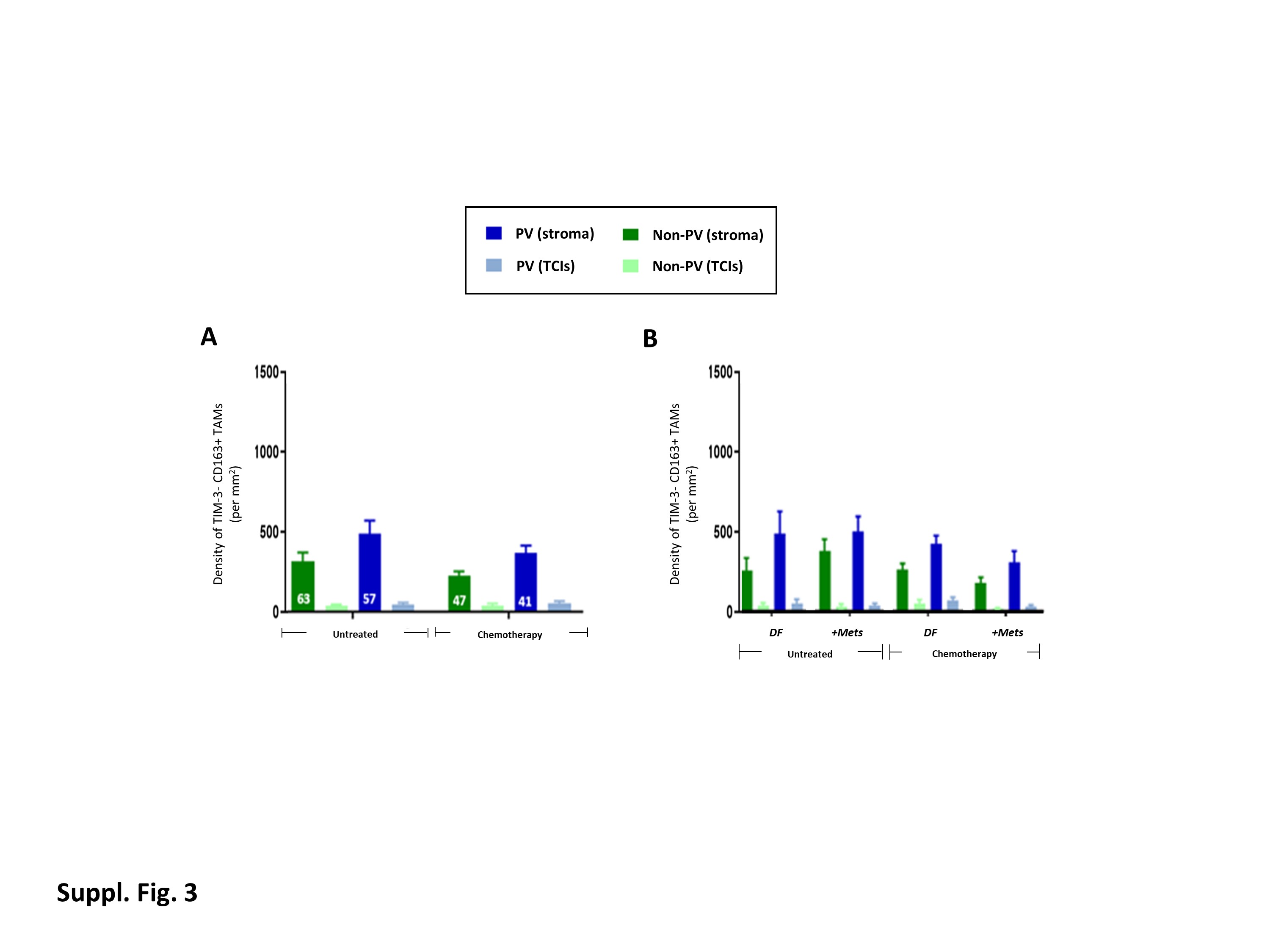

Supplement: Supplementary Figure 3 — Frequency and distribution of TIM-3-CD163+TAMs. (A) TIM-3-CD163+ TAMs were present throughout the stroma in untreated and NAC-treated tumors. The white figures at the base of each bar are the % of CD163+ TAMs in each group that were TIM-3-. (B) The density of this TAM subset did not correlate with metastasis. [NB. in all groups, stromal PV and non-PV groups were significantly higher than corresponding groups in TCIs, asterices not shown for clarity]. P<0.05. [file Image_3.jpg]

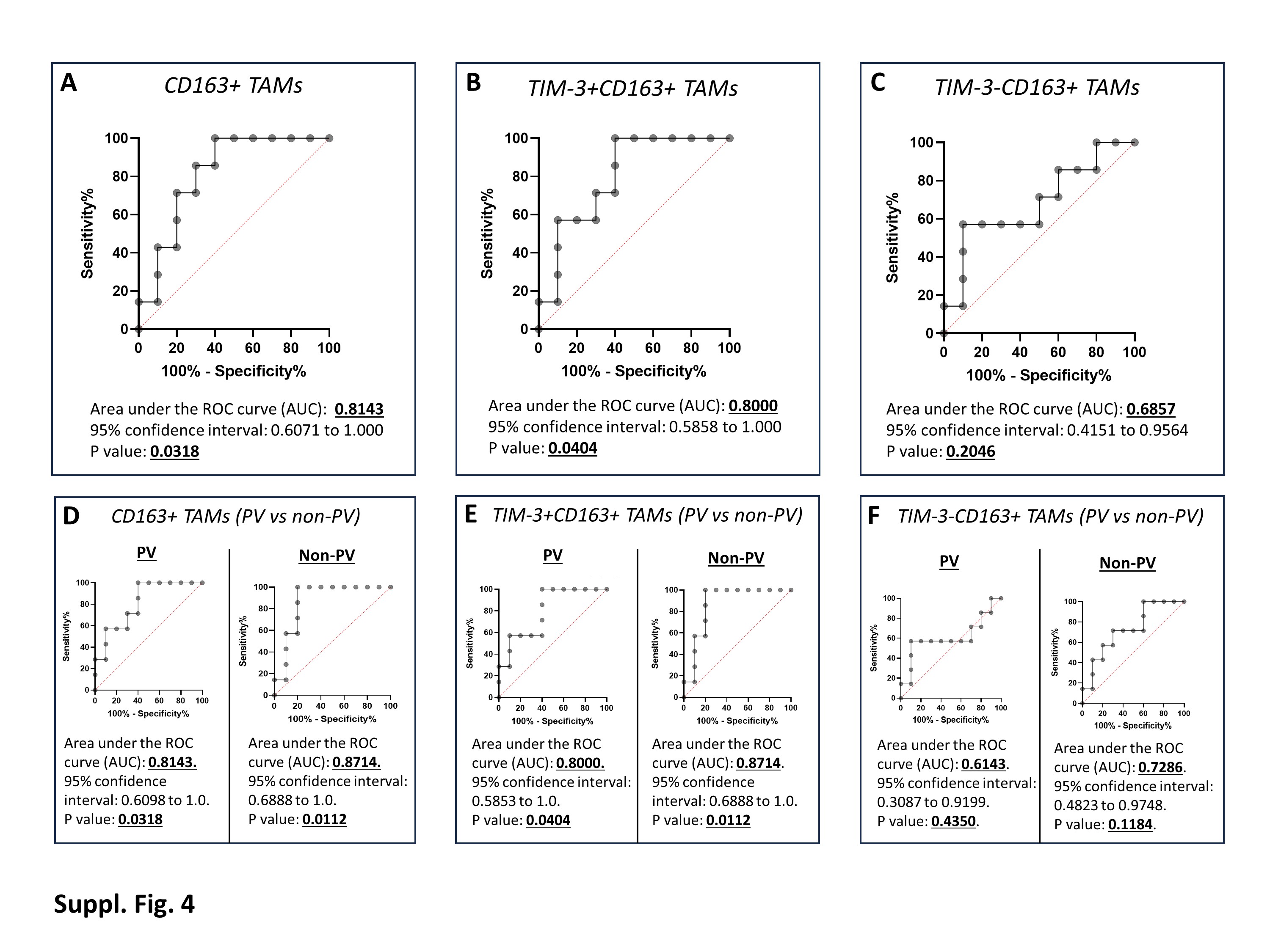

Supplement: Supplementary Figure 4 — Receiver Operating Characteristic (ROC) curve analysis of the ability of three stromal TAM subsets to predict the development of metastasis in NAC-treated patients within 3 years of surgical removal of primary tumors (ROC analysis of data at the level of whole tumor sections). (A) all CD163+ TAMs; (B) all TIM3-3+CD163+ TAMs; (C) all TIM-3-CD163+ TAMs; (D) PV v non-PV CD163+ TAMs; (E) PV v non-PV TIM-3+CD163+ TAMs; (F) TIM-3-CD163+ TAMs. [file Image_4.jpg]

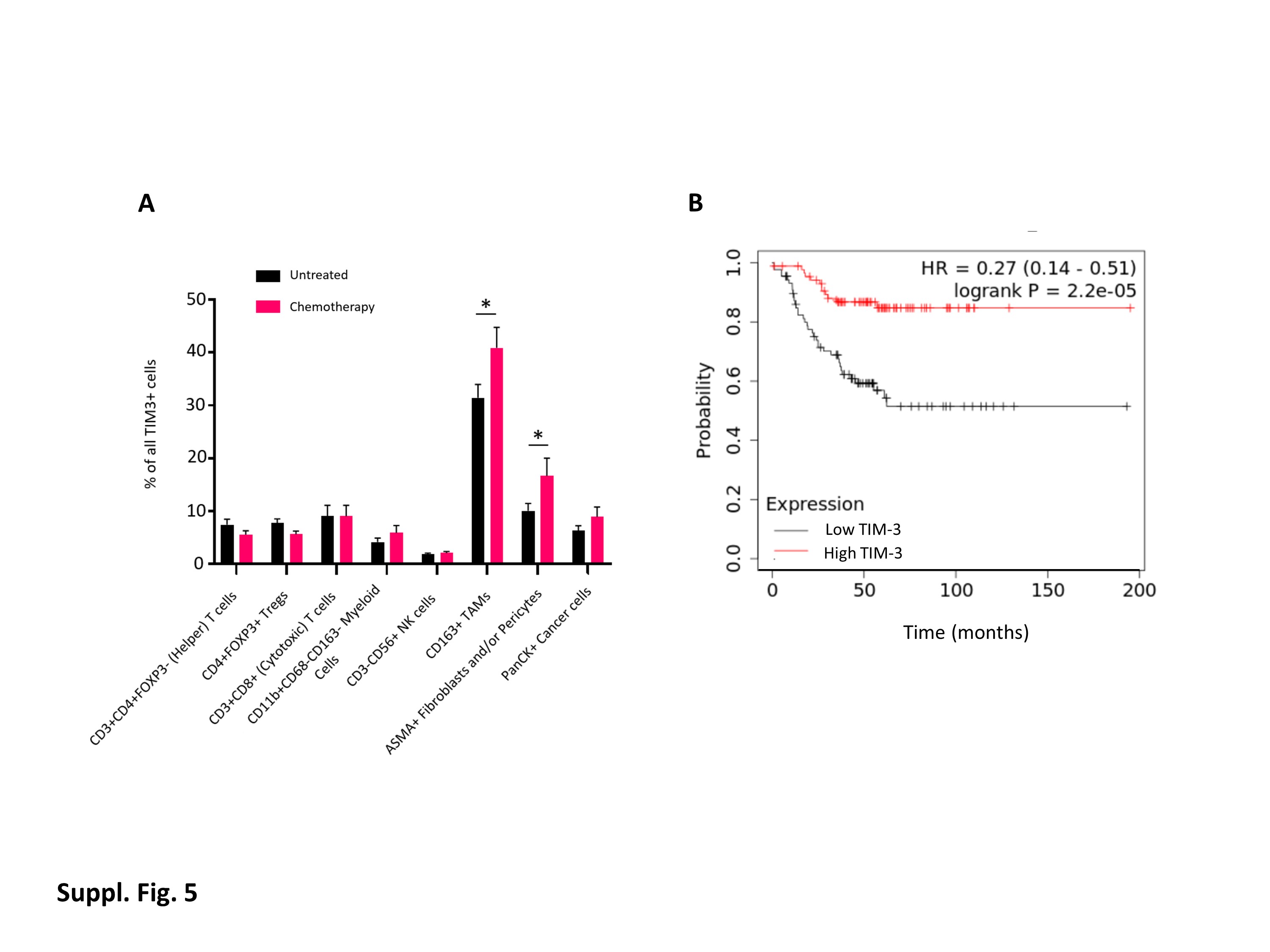

Supplement: Supplementary Figure 5 — Expression of TIM-3 in human TNBC: correlation with relapse free survival after NAC. (A) CD163+ TAMs and alpha smooth muscle actin-positive cells (‘ASMA’; pericytes and fibroblasts) are the predominant cell types expressing TIM-3 - and the proportion of these cells expressing this cell surface protein increases after NAC. ASMA = alpha smooth muscle actin. *P<0.05. (B) Significant correlation between TIM-3 expression levels in 153 TNBCs (taken from women administered NAC) and DFS (using online dataset: http://kmplot.com/analysis/index.php?p=service&cancer=breast#) (33). [file Image_5.jpg]

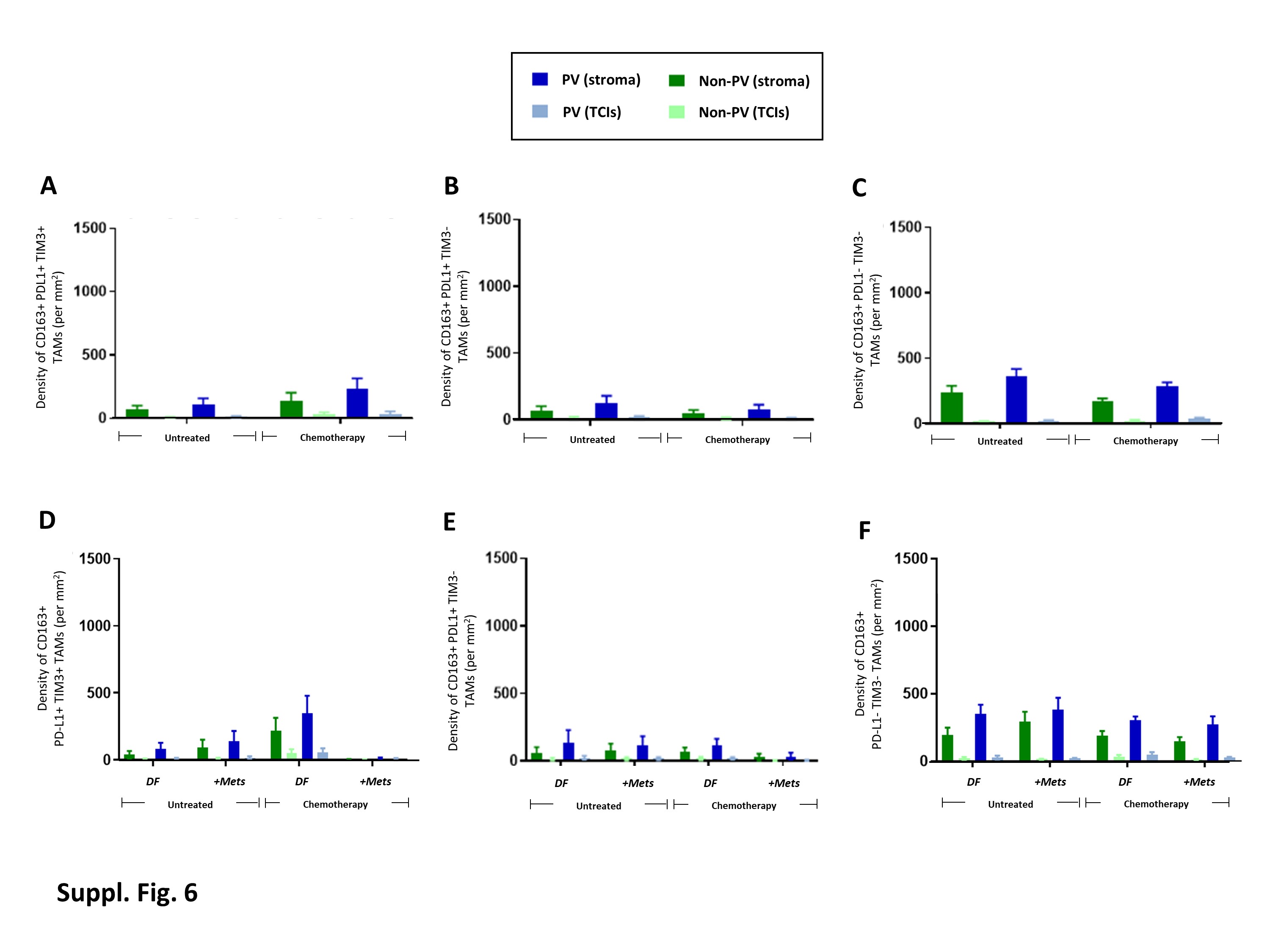

Supplement: Supplementary Figure 6 — No effect of NAC on various CD163+ TAM subsets. Three CD163+ TAM subsets (A, D) PD-L1+TIM-3+; (B, E) PD-L1+TIM-3-; (C, F). PD-L1-TIM-3) were evenly distributed throughout the stroma of tumors at significantly higher density than in corresponding areas of TCIs (asterices not shown). There was no effect of NAC on the density or distribution of these cells, nor did they correlate with metastasis. (ns = not statistically significant). [file Image_6.jpg]

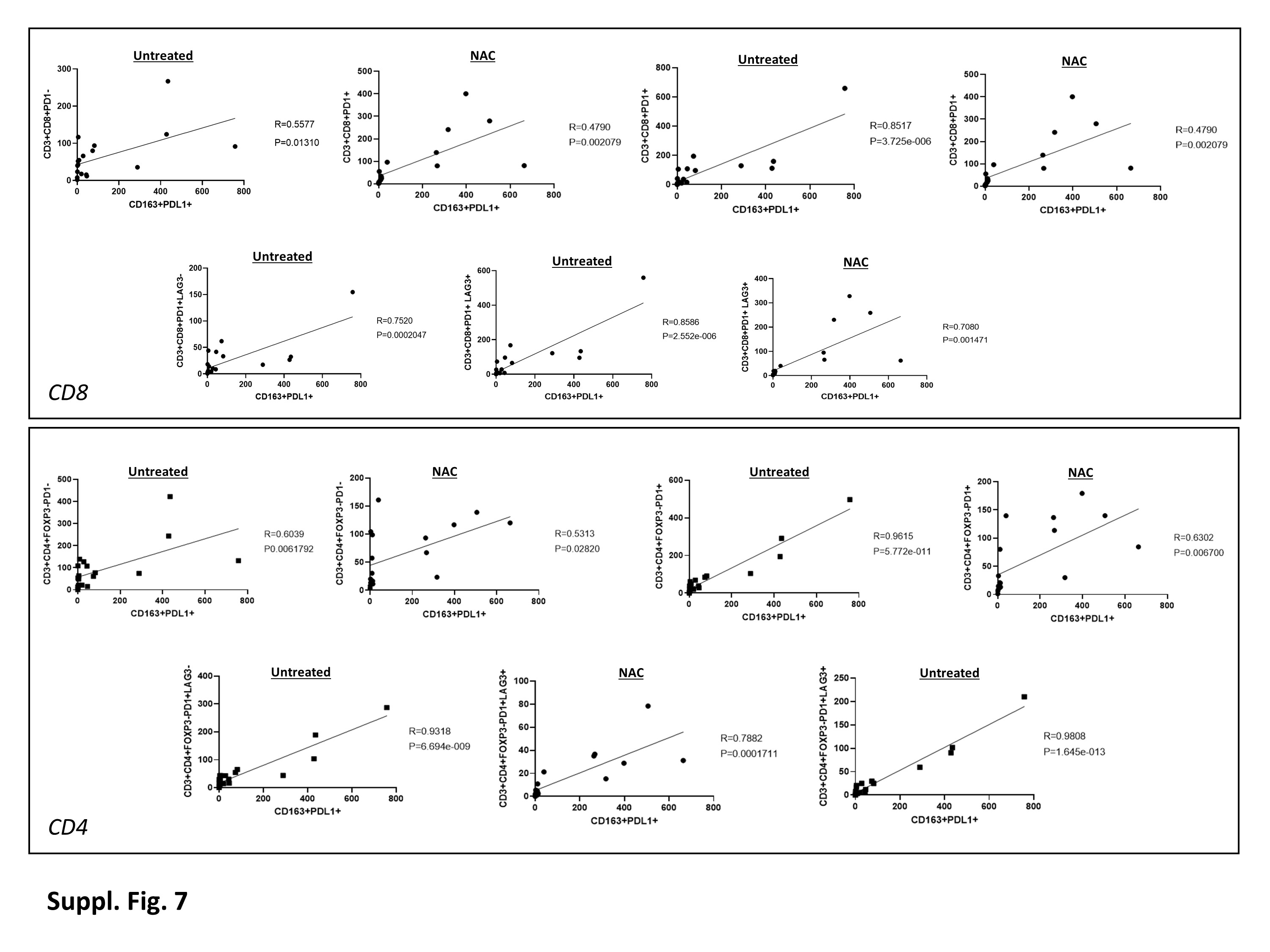

Supplement: Supplementary Figure 7 — Correlation graphs for significantly different comparisons seen in Supplementary Table 2 . R = correlation coefficient. [file Image_7.jpg]
