# Supplementary material for: Changes in the immune landscape of TNBC after neoadjuvant chemotherapy: correlation with relapse
Source: Front Immunol. 2023 Nov 9;14:1291643. doi: 10.3389/fimmu.2023.1291643 (PMC10715438; doi:10.3389/fimmu.2023.1291643)
Supplement: Supplementary Table 1 — Clinical parameters for the four patent groups analysed in this study. Parentheses indicate percentage data. NAC = neoadjuvant chemotherapy (3-8 cycles). DF = DF (no metastasis within 3 years of primary surgery), ‘+Mets’ = developed metastases within 3 years. FEC = 5 fluorouracil, epirubicin, cyclophosphamide. FEC-T - 5 = fluorouracil, epirubicin, cyclophosphamide, docetaxel. ‘Other’ NACs included FEC-T & Carboplatin, EC (epirubicin cyclophosphamide) and ECD (epirubicin cyclophosphamide & docetaxel). [file Table_1.pdf]

Suppl. Table 1

	Untreated - DF	Untreated + Mets	NAC - DF	NAC+ Mets
**Age at diagnosis**				
21-39	2 (22.2)	2 (20)	1 (10)	2 (28.6)
40-59	2 (22.2)	2 (20)	7 (70)	3 (42.8)
60-79	3 (33.3)	6 (60)	2 (20)	2 (28.6)
80-99	2 (22.2)	0 (0)	0 (0)	0 (0)
**Tumour size**				
T1	4 (44.4)	2 (20)	2 (20)	2 (28.6)
T2	5 (55.5)	7 (70)	7 (70)	5 (71.5)
T3	0 (0)	1 (10)	1 (10)	1 (14.3)
**Tumour grade**				
2	1 (11.1)	2 (20)	0 (0)	0 (0)
3	8 (88.8)	8(80)	10 (100)	7 (100)
**Time between end of treatment and surgery (days)**				
<30	n/a	n/a	3 (30)	2 (28.6)
30-40	n/a	n/a	4 (40)	3 (42.8)
>40	n/a	n/a	3 (30)	2 (28.6)
**Lymph node status**				
Involved	3 (33.3)	7 (70)	4 (40)	7 (100)
Not involved	5 (55.5)	2 (20)	5 (50)	0 (0)
Not sampled	1 (11.1)	1 (10)	1 (10)	0 (0)
**Neoadjuvant therapy received**				
FEC-T	0 (0)	0 (0)	7 (70)	3 (42.8)
FEC	0 (0)	0 (0)	2 (20)	1 (14.3)
Other	0 (0)	0 (0)	1 (10)	3 (42.8)
None	9(100)	10 (100)	0 (0)	0 (0)
**Metastases within 3-year follow-up?**				
Yes	0 (0)	10 (100)	0 (0)	7 (100)
No	9(100)	0 (0)	10 (100)	0 (0)
**Adjuvant therapy received**				
Radiotherapy only	1 (11.1)	0 (0)	5 (50)	6 (85.6)
Radiotherapy & Chemotherapy	6 (66.6)	6 (60)	5 (50)	1 (14.3)
Chemotherapy only	1 (11.1)	2 (20)	0 (0)	0 (0)
None	1 (11.1)	1 (10)	0 (0)	0 (0)
No data	0 (0)	1 (10)	0 (0)	0 (0)
**Adjuvant chemotherapy received**				
Carboplatin	0 (0)	0 (0)	2 (20)	1 (14.3)
Carboplatin & Gemcitabine	0 (0)	0 (0)	2 (20)	2 (28.6)
Docetaxel	0 (0)	0 (0)	0 (0)	1 (14.3)
FEC-T	5 (55.5)	4 (40)	0 (0)	0 (0)
Other	2 (22.2)	4 (40)	1 (10)	2 (28.6)
None	2 (22.2)	1 (10)	5 (50)	1 (14.3)
No data	0 (0)	1 (10)	0 (0)	0 (0)
