# Supplementary material for: Changes in the immune landscape of TNBC after neoadjuvant chemotherapy: correlation with relapse
Source: Front Immunol. 2023 Nov 9;14:1291643. doi: 10.3389/fimmu.2023.1291643 (PMC10715438; doi:10.3389/fimmu.2023.1291643)
Supplement: Supplementary Table 2 — Correlation of the overall density of PD-L1+ CD163+TAMs with CD4+ T cells, CD8+ T cells and Tregs. [file Table_2.pdf]

Suppl. Table 2

Correlation	*Untreated*		*Chemotherapy*	
	R	*p value*	*R*	*P value*
Cytotoxic (CD8+) T Cells				
CD163+PDL1- TAMs v CD3+CD8+PD1-	*0.27*	*NS*	*0.20*	*NS*
CD163+PDL1- TAMs v CD3+CD8+PD1+	*- 0.15*	*NS*	*- 0.002*	*NS*
CD163+PDL1+ TAMs v CD3+CD8+PD1-	*0.56*	*0.01*	*0.4*	*NS*
CD163+PDL1+ TAMs v CD3+CD8+PD1+<br>*…..LAG3- (active)*<br>*…………LAG3+ (exhausted)*	*0.84*<br>*0.75*<br>*0.86*	*<0.0001*<br>*<0.0001*<br>*<0.0001*	*0.48*<br>*0.52*<br>*0.70*	*0.002*<br>*0.03*<br>*0.001*
Helper (CD4+FOXP3-) T Cells				
CD163+PDL1- TAMs v CD3+CD4+FOXP3-PD1-	0.15	NS	0.22	NS
CD163+PDL1- TAMs v CD3+CD4+FOXP3-PD1+	-0.15	NS	0.23	NS
CD163+PDL1+ TAMs v CD3+CD4+FOXP3-PD1-	0.60	0.006	0.53	0.03
CD163+PDL1+TAMs v CD3+CD4+FOXP3-PD1+<br>*…LAG3- (active)*<br>*……….LAG3+ (exhausted)*	*0.96*<br>*0.93*<br>*0.98*	*<0.0001*<br>*<0.0001*<br>*<0.0001*	*0.63*<br>*0.46*<br>*0.79*	*0.007*<br>*NS*<br>*<0.0001*
Regulatory (CD4+FOXP3+) T cells				
CD163+PDL1- TAMs v CD3+CD4+FOXP3+PD1-	0.24	NS	0.06	NS
CD163+PDL1- TAMs v CD3+CD4+FOXP3+PD1+	-0.25	NS	0.42	NS
CD163+PDL1+ TAMs v CD3+CD4+FOXP3+PD1-	0.73	0.0004	0.61	0.009
CD163+PDL1+ TAMs v CD3+CD4+FOXP3+PD1+	0.85	<0.0001	0.65	0.005
